# Macrophage polarization during *Streptococcus agalactiae* infection is isolate specific

**DOI:** 10.3389/fmicb.2023.1186087

**Published:** 2023-05-04

**Authors:** Larisa Janžič, Jernej Repas, Mojca Pavlin, Špela Zemljić-Jokhadar, Alojz Ihan, Andreja Nataša Kopitar

**Affiliations:** ^1^Department of Cell Immunology, Institute of Microbiology and Immunology, Faculty of Medicine, University of Ljubljana, Ljubljana, Slovenia; ^2^Institute of Biophysics, Faculty of Medicine, University of Ljubljana, Ljubljana, Slovenia; ^3^Group for Nano and Biotechnological Applications, Faculty of Electrical Engineering, University of Ljubljana, Ljubljana, Slovenia

**Keywords:** macrophages, *Streptococcus agalactiae*, immunometabolism, immunophenotyping, phagocytosis, cytotoxicity

## Abstract

**Introduction:**

*Streptococcus agalactiae* (Group B *Streptococcus*, GBS), a Gram-positive commensal in healthy adults, remains a major cause of neonatal infections, usually manifesting as sepsis, meningitis, or pneumonia. Intrapartum antibiotic prophylaxis has greatly reduced the incidence of early-onset disease. However, given the lack of effective measures to prevent the risk of late-onset disease and invasive infections in immunocompromised individuals, more studies investigating the GBS-associated pathogenesis and the interplay between bacteria and host immune system are needed.

**Methods:**

Here, we examined the impact of 12 previously genotyped GBS isolates belonging to different serotypes and sequence types on the immune response of THP-1 macrophages.

**Results:**

Flow cytometry analysis showed isolate-specific differences in phagocytic uptake, ranging from 10% for isolates of serotype Ib, which possess the virulence factor protein β, to over 70% for isolates of serotype III. Different isolates also induced differential expression of co-stimulatory molecules and scavenger receptors with colonizing isolates inducing higher expression levels of CD80 and CD86 compared to invasive isolates. In addition, real-time measurements of metabolism revealed that macrophages enhanced both glycolysis and mitochondrial respiration after GBS infection, with isolates of serotype III being the most potent activators of glycolysis and glycolytic ATP production. Macrophages also showed differential resistance to GBS-mediated cell cytotoxicity as measured by LDH release and real-time microscopy. The differences were evident both between serotypes and between isolates obtained from different specimens (colonizing or invasive isolates) demonstrating the higher cytotoxicity of vaginal compared with blood isolates.

**Conclusions:**

Thus, the data suggest that GBS isolates differ in their potential to become invasive or remain colonizing. In addition, colonizing isolates appear to be more cytotoxic, whereas invasive isolates appear to exploit macrophages to their advantage, avoiding the immune recognition and antibiotics.

## 1. Introduction

*Streptococcus agalactiae* (Group B *Streptococcu*s, GBS) is a Gram positive, encapsulated, β-hemolytic commensal of the lower genital and gastrointestinal tracts in 15–30% of healthy adults (Armistead et al., 2019), but it also remains a leading cause of neonatal sepsis, pneumonia and meningitis (Landwehr-Kenzel and Henneke, 2014). It is usually transmitted from colonized mother to the fetus *in utero* (so called ascending infection) or to a neonate during birth. In addition to the vertical transmission, horizontal transmission later in life is also possible (Sagar et al., 2013), making GBS a serious cause of invasive disease in pregnant women and in elderly or immunocompromised people (Pietrocola et al., 2018). GBS causes two types of neonatal diseases: (a) early-onset disease (EOD), which occurs within the first week of life and typically presents as a sepsis or pneumonia, and (b) late-onset disease (LOD), which develops between day 7 and 3 months of age and usually presents as a bloodstream infection leading to meningitis (Korir et al., 2017b). During delivery, colonized women are given intrapartum antibiotic prophylaxis (IAP) which has greatly decreased the incidence of EOD but not LOD (Landwehr-Kenzel and Henneke, 2014). Because IAP is also ineffective for ascending infections, new therapies are needed to effectively prevent and treat GBS infections. To develop such therapies, it is imperative to gain a better insight into the interactions of GBS with the host immune system.

Based on capsular polysaccharides GBS can be classified into 10 different serotypes: Ia, Ib, II – IX, with serotypes Ia, Ib, III, and V most often causing the disease (Pietrocola et al., 2018). Using multi-locus sequence typing (MLST) which analyses allelic variation in 7 housekeeping genes, isolates are further classified into sequence types (ST) and clustered into clonal complexes (CC) based on sequence similarities (Perme et al., 2020). MLST technique revealed that GBS isolates with the same STs may belong to different serotypes (Shabayek and Spellerberg, 2018). GBS isolates therefore vary at both genotypic and phenotypic levels and this diversity is thought to play a key role in the severity of GBS disease (Flaherty et al., 2021).

Although in most cases GBS persists as a part of the microbiota and does not cause infections, its virulence factors allow it to switch from commensal to invasive pathogen. However, the trigger for the switch into invasive phenotype is not yet fully understood (Shabayek and Spellerberg, 2018). The following steps are required for the development of invasive disease: (1) intestinal colonization, (2) translocation across the intestinal barriers, and (3) evasion of the immune system (Landwehr-Kenzel and Henneke, 2014).

GBS contains several virulence factors crucial for adhesion to the extracellular matrix (ECM) or host cell (Pietrocola et al., 2018). Some of these (the GBS hypervirulent adhesin HvgA) also allow the crossing of both the intestinal and the blood–brain barrier (Landwehr-Kenzel and Henneke, 2014). Another important virulence factor for GBS adhesion are the pili – extensions of the bacterial cell surface that are anchored to the cell wall (Shabayek and Spellerberg, 2018). In GBS, three pilus islands were identified: PI-1, PI-2A and PI-2B (Landwehr-Kenzel and Henneke, 2014).

GBS activates phagocytes through interactions of bacterial lipoproteins with Toll-like receptors (TLR) 2 and 6 and elicits the production of pro-inflammatory cytokines. Beside lipoproteins, GBS nucleic acids also strongly activate inflammatory genes in phagocytes (Landwehr-Kenzel and Henneke, 2014). In addition to producing inflammatory cytokines, ROS and NO (Korir et al., 2017b), macrophages (such as neutrophils) secrete macrophage extracellular traps (METs), which play a role in capturing and killing bacteria (Doster et al., 2018). However, GBS successfully evades immune recognition by expressing the polysaccharide capsule, which contains a terminal sialic acid that is also present on the surface of vertebrate cells and allows GBS to mimic host cells (Wessels et al., 1989). Moreover, some GBS isolates express β-protein, a surface protein that binds to the complement inhibitor FH (Pietrocola et al., 2018), thereby preventing the deposition of the C3b component of complement and opsonophagocytosis via the alternative pathway of complement activation (Korir et al., 2017b). It also binds to the Fc fragment of IgA antibodies and to Siglec-5, which is an inhibitory receptor for phagocytosis thereby attenuating innate immunity and promoting bacterial survival (Dobrut and Brzychczy-Wloch, 2021). In addition to the β-protein, complement activation is also influenced by other virulence factors, such as the GBS immunogenic bacterial adhesion protein (BibA) and streptococcal C5a peptidase (ScpB) (Pietrocola et al., 2018). However, studies have shown that GBS, once phagocytosed, can survive in macrophages due to their ability to inactivate the production of ROS and NO synthesis (Poyart et al., 2001) and resist host antimicrobial proteins (Hamilton et al., 2006). One of the major virulence factors produced by GBS is also β-hemolysin/cytolysin toxin, an ornithine rhamnolipid that promotes invasion across host cell barriers and pro-inflammatory cytokine response. Moreover, it also causes lysis of erythrocytes and macrophage pyroptosis (Whidbey et al., 2015) as activation of the NLRP3 inflammasome by GBS is dependent on the expression of β-hemolysin/cytolysin (Costa et al., 2012).

Neonates are highly susceptible to invasive pathogens due to their very underdeveloped and immunologically inexperienced adaptive immune system, raising the relative importance of innate immunity for protection against microbes (Kumar and Bhat, 2016). As phagocytes, macrophages are the first line of defense involved in pathogen recognition, phagocytosis and destruction, recruitment of other immune cells to the site of infection, maintenance of tissue homeostasis and immunosuppression (Zhang and Wang, 2014; Geeraerts et al., 2017; Galli and Saleh, 2020). Stimuli and signals from the microenvironment dictate the so-called macrophage polarization determining their phenotype and functional response (Shapouri-Moghaddam et al., 2018). Roughly, they can be divided into classically activated M1 and alternatively activated M2 macrophages (Benoit et al., 2008). Upon stimulation with Th1 cytokines IFN-γ and TNF-α or bacterial LPS, macrophages polarize toward M1 phenotype, producing high levels of pro-inflammatory cytokines and generating reactive oxygen species (ROS) through activation of the NADPH system (Geeraerts et al., 2017; Shapouri-Moghaddam et al., 2018). They produce ATP mainly by enhanced glycolysis and the pentose phosphate pathway (PPP) (Nonnenmacher and Hiller, 2018), express high levels of co-stimulatory molecules CD80 and CD86 and possess bactericidal and anti-tumor activity (Geeraerts et al., 2017; Takiguchi et al., 2021). On the other hand, upon stimulation with Th2 cytokines IL-4 and IL-13, macrophages obtain an anti-inflammatory M2 phenotype. They have a potent phagocytosis capacity and are involved in immunosuppression, tissue remodeling and tumor progression (Shapouri-Moghaddam et al., 2018). M2 macrophages generate ATP mainly through oxidative phosphorylation (OxPhos) (Geeraerts et al., 2017), produce anti-inflammatory cytokines, and express high levels of scavenger receptors such as CD163 and CD206 (Shapouri-Moghaddam et al., 2018; Takiguchi et al., 2021). However, the M1/M2 classification does not reflect the heterogeneity of macrophages *in vivo* and several studies have shown that these categories represent the extremes of the whole spectrum of phenotypes *in vivo* (Diskin and Palsson-McDermott, 2018). Importantly, as the M1/M2 classification applies mainly to macrophages stimulated with the aforementioned stimuli, it therefore does not adequately describe macrophages stimulated with live bacteria (Traven and Naderer, 2019). In addition, gram positive bacteria lack LPS, which makes stimulations with live bacteria even more crucial to better understand the impact of infection on physiologically relevant immune response of macrophages.

Since the disease severity depends on both the GBS isolate and the immune status of the individual, we explored possible isolate-specific differences in the immune response to identify patterns that would enable us to determine which isolates are more invasive. To this end, we investigated the influence of 12 different GBS isolates varying in capsular serotype, ST, CC, virulence factors and the severity of the disease on phagocytosis and expression of macrophage phenotype markers. In addition, we determined GBS mediated cell cytotoxicity and the macrophage metabolic profile (rate of glycolysis and mitochondrial respiration) after infection with live GBS isolates.

Since there is still much unknown about the GBS-associated pathogenesis, the aim of the study is to provide better insight into the immune response of macrophages to various GBS isolates, especially since innate immune system is of particular importance for neonates.

## 2. Materials and methods

### 2.1. Bacterial isolates

GBS isolates were obtained from infected newborns with invasive disease or colonized pregnant women between 2001 and 2018, were kindly provided by Samo Jeverica and are kept at the Institute of Microbiology and Immunology, Faculty of Medicine, University of Ljubljana. All isolates were previously genotyped by Perme et al. (2020). Twelve different isolates, belonging to 6 different serotypes (Ia, Ib, II – V), 10 different STs and 5 different CCs were selected. Isolates also differ in virulence factors, Clinical presentation (invasive or colonizing) and the specimen from which they were obtained (blood, cerebrospinal fluid, or vagina/vagina-rectum). (Table 1). All the selected isolates contain the following virulence factors: ScpB, Lmb, BibA, FbsA, FbsB. However, they differ by pilus type, Srr and Alp proteins, and the presence or absence of HvgA or β-proteins. These characteristics are summarized in Table 1.

**Table 1:** Characteristics of *Streptococcus agalactiae* isolates used in the study.

Isolate id	Serotype	Sequence type (ST)	Clonal complex (CC)	Clinical presentation	Specimen	Pilus island	Virulence factors
229	Ia	ST-23	CC-23	LOD	CSF	PI-2A	Alp1
9427	Ia	ST-144	CC-23	Colonization	Vagina-rectum	PI-2A	Rib protein
203	Ib	ST-8	CC-12	EOD	Blood	PI-1-2A	α-protein, β-protein
10276	Ib	ST-9	CC-12	Colonization	Vagina	PI-1-2A	α-protein, β-protein
211	II	ST-28	CC-19	EOD	Blood	PI-1-2A	Rib protein
7339	II	ST-12	CC-12	Colonization	Vagina	PI-1-2A	α-protein, β-protein
231	III	ST-17	CC-17	EOD	Blood	PI-1-2B	Rib protein, HvgA
9731	III	ST-17	CC-17	Colonization	Vagina	PI-1-2B	Rib protein, HvgA
8422	IV	ST-291	CC-17	Colonization	Vagina-rectum	PI-1-2B	Rib protein, HvgA
6	V	ST-1	CC-1	EOD	CSF	PI-1-2A	R28 protein, delta 357 ScpB deletion
104	V	ST-1	CC-1	EOD	Blood	PI-1-2A	R28
123	V	ST-1	CC-1	EOD	Blood	PI-2A	α-protein

Isolates were grown from frozen stocks on blood agar plates at 37°C in ambient air overnight. The following day, a single bacterial colony was subcultured from blood agar plates in Todd-Hewitt broth (THB) and incubated overnight under aerobic shaking conditions at 37°C in ambient air.

The purity of the bacterial cultures was regularly tested by inoculating the overnight cultures from THB onto blood agar plates and examining the colonies the next day.

### 2.2. Cell line and macrophage differentiation

The acute monocytic human leukemia cell line THP-1 cells (ATCC^®^ TIB-202^™^) were cultured in RPMI 1640 medium supplemented with 10 mM HEPES, 2 mM L-glutamine, 25 mM D-glucose, 1 mM sodium pyruvate (Gibco, Thermo Fisher Scientific) and 10% fetal bovine serum (Sigma-Aldrich) at 37°C with 5% CO_2_ and were passaged every 3–4 days. Cells were differentiated into macrophages by incubation with 100 nM phorbol 12-myristate 13-acetate (PMA; Sigma-Aldrich) for 3 days, followed by 5-day rest in a medium without PMA. The differentiation protocol was selected based on preliminary results in which THP-1 monocytes were differentiated into macrophages with different PMA concentrations (30, 100, and 162 nM) and for different periods of time (24 h and 1 day rest, 72 h and 1 day rest and 24 or 72 h and 5 day rest), and then the expression of CD11c (CD11c PE, BD Pharmigen), CD14 (CD14 PerCP-Cy5.5, BD Pharmigen), CD16 (CD16 APC, BD Pharmigen), CD68 (CD68 FITC, BioLegend), CD163 (CD163 PE, Invitrogen) and viability (7-AAD, BD) were measured by flow cytometry and confocal microscopy.

### 2.3. Opsonization and FITC labeling of GBS

Prior to bacterial infection, GBS isolates were harvested by centrifugation, washed in sterile phosphate-buffered saline (PBS), and opsonized in human serum for 30 min at 37°C, with shaking. For the phagocytosis assay, GBS isolates were labeled with 0.1 mg/ml FITC in 0.1 M NaHCO_3_ for 1 h at room temperature, washed 4 times in PBS and resuspended in antibiotic-free cell culture medium.

### 2.4. Infection of THP-1 macrophages with GBS isolates

Differentiated THP-1 macrophages were washed in PBS. GBS cultures were harvested as described in 2.3 and resuspended in antibiotic-free cell culture medium. Bacterial density was measured using the DensiCHEK Plus Instrument (BioMerieux) optical densitometer. The optical density of the bacterial suspension was set to OD_580_ = 0.5, which corresponds to a concentration of 1.5 × 10^8^ CFU/ml. The accuracy of the densitometer was also regularly checked by appropriate serial dilutions and CFU quantification.

THP-1 macrophages were stimulated with live GBS at a multiplicity of infection (MOI) of 10 bacteria per host cell for 3 h at 37°C with 5% CO_2_ unless otherwise noted. The calculation of MOI was verified using CFU quantification.

### 2.5. Flow cytometry analysis

#### 2.5.1. Phagocytosis assay

To determine bacterial phagocytosis, THP-1 cells were seeded in a 24-well plate at a density of 4 × 10^5^ cells/ml, differentiated into macrophages and stimulated with FITC-labeled GBS isolates for 3 h at 37°C as described in 2.3 and 2.4. A negative control was stimulated with live bacteria on ice. The phagocytosis assay was performed with optimized PHAGOTEST kit (Glycotope Biotechnology GmbH). Briefly, phagocytosis was stopped by placing the plate on ice. Stimulated macrophages were washed with PBS, detached, and then a quenching solution was added to suppress the fluorescence of extracellular bacteria. Cells were fixed and DNA staining solution was added just before the measurement. Cells were analyzed on FACSCanto II flow cytometer (Becton Dickinson), using BD FACSDiva v8.0.1 software (Becton Dickinson). The percentage of cells that phagocytosed the FITC labeled GBS isolates was determined based on the gate of the negative control sample in the FITC fluorescence histogram.

#### 2.5.2. Expression of M1 and M2 macrophage polarization markers

Expression of M1 and M2 macrophage phenotype markers was analyzed using a BD FACSCanto II flow cytometer with BD FACSDiva software. Briefly, THP-1 cells were seeded in a 12-well plate at a density of 4 × 10^5^ cells/ml, differentiated into macrophages and stimulated with GBS isolates as described in 2.3 and 2.4. Non-stimulated cells were used as a negative control. After 3 h, extracellular bacteria were killed by washing the unattached bacteria and adding fresh RPMI 1640 supplemented with 2% FBS, 100 μg/ml gentamicin (Krka), and 5 μg/ml penicillin G (Sandoz). Cells were then incubated for an additional 72 h at 37°C and 5% CO_2_ to achieve maximal phenotype markers expression. Following incubation, cells were washed, detached, and stained extracellularly with monoclonal antibodies (mAbs). A combination of mAbs conjugated with fluorochromes against the following cell surface molecules was used: CD64-FITC (BioLegend), CD80-APC (MACS), CD86-Viol700 (Becton Dickinson), CD163-PE (eBioscience) and CD206-APC (MACS). Labeled macrophages were washed and BD Cytofix^™^ was added for subsequent intracellular staining of CD68, labeled with FITC (BioLegend). Normalized median fluorescence intensities (nMFIs) were determined after dividing the data by the fluorescence values of the negative control.

### 2.6. Cytotoxicity assay

Bacterial cell-mediated cytotoxicity was detected by measuring the amount of lactate dehydrogenase (LDH) in the culture supernatant. The CytoTox 96 Non-Radioactive Cytotoxicity Assay Kit (Promega) was used according to the manufacturer’s instructions. THP-1 cells were seeded in a 96-well plate at a density of 4 × 10^5^ cells/ml, differentiated into macrophages as described in 2.2 and stimulated with 100 μl aliquots of bacterial suspensions at a MOI of 10:1 and 20:1 for 3 h at 37°C. The plate was then centrifuged, and supernatants were collected in a new plate. Macrophages to which lysis solution was added served as a positive, high control (100% cytotoxicity), whereas non-infected macrophages served as a negative, low control. LDH release was determined by measuring absorbance at 490 nm using the Cytation 5 Multi-Mode Reader (BioTek). The percentage of LDH release was calculated by using the supernatant of lysed cells as 100% LDH release and the supernatant of untreated cells as a negative control.

### 2.7. Seahorse extracellular flux analysis

#### 2.7.1. Real-time bioenergetics assay

THP-1 cells were seeded in a 24-well Seahorse cell culture plate at a density of 1.5 × 10^5^ cells per well and differentiated into macrophages as described in 2.2. Differentiated THP-1 macrophages were stimulated with GBS isolates at a MOI of 10:1 for 3 h at 37°C. Non-stimulated macrophages were used as a negative control. After 3 h, macrophages were washed with PBS and fresh RPMI 1640 supplemented with 2% FBS, 100 μg/ml gentamicin, and 5 μg/ml penicillin G was added for another hour to kill extracellular bacteria. RPMI with antibiotics was then replaced with RPMI 1640 based Seahorse XF assay medium (Agilent) supplemented with 5.6 mM glucose and 2 mM glutamine, and the plate was incubated for an additional 45–55 min in a CO_2_-free incubator at 37°C. Basal oxygen consumption rate (OCR) and extracellular acidification rate (ECAR) were measured in real-time, for 10 sequential measurements to capture any dynamic changes of metabolism following bacterial stimulation. The eleventh basal measurement was considered the basal metabolic phenotype and was selected based on previous pilot experiments. After this, 1.5 μM oligomycin (an ATP synthase (complex V) inhibitor that allows calculation of mitochondrial O_2_ consumption coupled to ATP production during OxPhos) and 0.5 μM antimycin-A plus 0.5 μM rotenone (complex III and complex I inhibitors that allow calculation of non-mitochondrial respiration driven by processes outside the mitochondria) (Van Den Bossche et al., 2015) were injected at designated time-points (Supplementary Figure S8) in the timing and sequence standard for the Seahorse Real-Time ATP Rate assay (Agilent, USA).

Non-mitochondrial oxygen consumption was measured after injection of rotenone and antimycin-A and normalized to control. To exclude the influence of bacterial metabolism, the metabolism of each isolate was measured at the beginning of the experiments and found to be minimal due to previous antibiotic treatment.

#### 2.7.2. Calculation of ATP production by seahorse real-time ATP rate assay

Seahorse Real-Time ATP Rate Assay was performed according to the manufacturer’s specifications (Agilent). ATP production through glycolysis (glycoATP) was calculated as glycolytic proton efflux rate according to equation: glycoATP (pmol ATP/min) = glycoPER (pmol H+/min) = basalPER (pmol H+/min) – MitoPER (pmol H+/min) = basalPER – (basal OCR – OCR after rotenone/antimycin A) * 0.6. The ATP production rate from oxidative phosphorylation (mitoATP) was calculated according to formula: mitoATP Production Rate (pmol ATP/min) = (basal OCR – OCR after oligomycin) (pmol O_2_/min) * 2 (pmol O/pmol O_2_) * P/O (pmol ATP/pmol O_2_) assuming a P/O ratio of 2.75 (Mookerjee et al., 2017).

### 2.8. Microscopy

#### 2.8.1. Fluorescence microscopy of macrophage morphology

Cells were seeded in Ibidi 2-well μ-slide (Ibidi GmbH), differentiated into macrophages and GBS isolates (isolates 203, 211, 231) were added for 3 h as described in 2.3 and 2.4. Non-infected macrophages were used as a control. Following infection, cells were washed and fresh RPMI 1640 supplemented with 2% FBS, 100 μg/ml gentamicin and 5 μg/ml penicillin G was added to kill extracellular bacteria. Cells were then incubated at 37°C and 5% CO_2_ for an additional 3 days to achieve maximal morphological changes. After incubation, cells were washed with RPMI and CellMask Green Plasma Membrane Stain (Invitrogen) was added at a dilution of 1:500 for 15 min to stain the membranes. Cells were visualized using a Nikon Eclipse TE2000-E confocal microscope with 60x objective. In addition, morphological changes were quantified in a blinded fashion and number of cells with altered morphology was enumerated.

#### 2.8.2. Real-time microscopy of macrophage-bacteria interactions

Cells were seeded in Delta T culture dish (Bioptechs) at a density of 4 × 10^5^ cells/ml and differentiated into macrophages by incubation with 100 nM PMA. Based on the results of previous experiments, three different GBS isolates were selected: isolate 231 (serotype III, ST-17), isolate 9427 (serotype Ia, ST-144) and isolate 203 (serotype Ib, ST-8). Bacteria were prepared as described in 2.3 and 2.4 and added to macrophages at a MOI of 10. The Delta T culture dish was placed in a confocal microscope (Nikon eclipse TE2000-E) and heated to 37°C throughout the experiment. In 3 h, 360 images were acquired, i.e., 1 image per 30 s with 600x magnification.

### 2.9. Statistical analysis

All data were tested for normal distribution with the Shapiro–Wilk or Kolmogorov–Smirnov test. Un-paired *t*-test, Mann–Whitney test, one-way ANOVA with *post-hoc* Tukey’s or Šidák’s multiple comparisons test or Kruskal-Wallis with *post-hoc* Dunn’s multiple comparisons test were used to analyze the statistical significance of the data. *p* ≤ 0.05 were considered statistically significant. Statistical analysis was performed using GraphPad Prism Software (Version 9.0, GraphPad Software Inc.). All graphs were drawn using GraphPad Prism Software or FlowJo Software (Version 10, Becton Dickinson).

## 3. Results

### 3.1. Phagocytosis of GBS is dependent on capsular serotype and certain virulence factors

**Figure 1:**
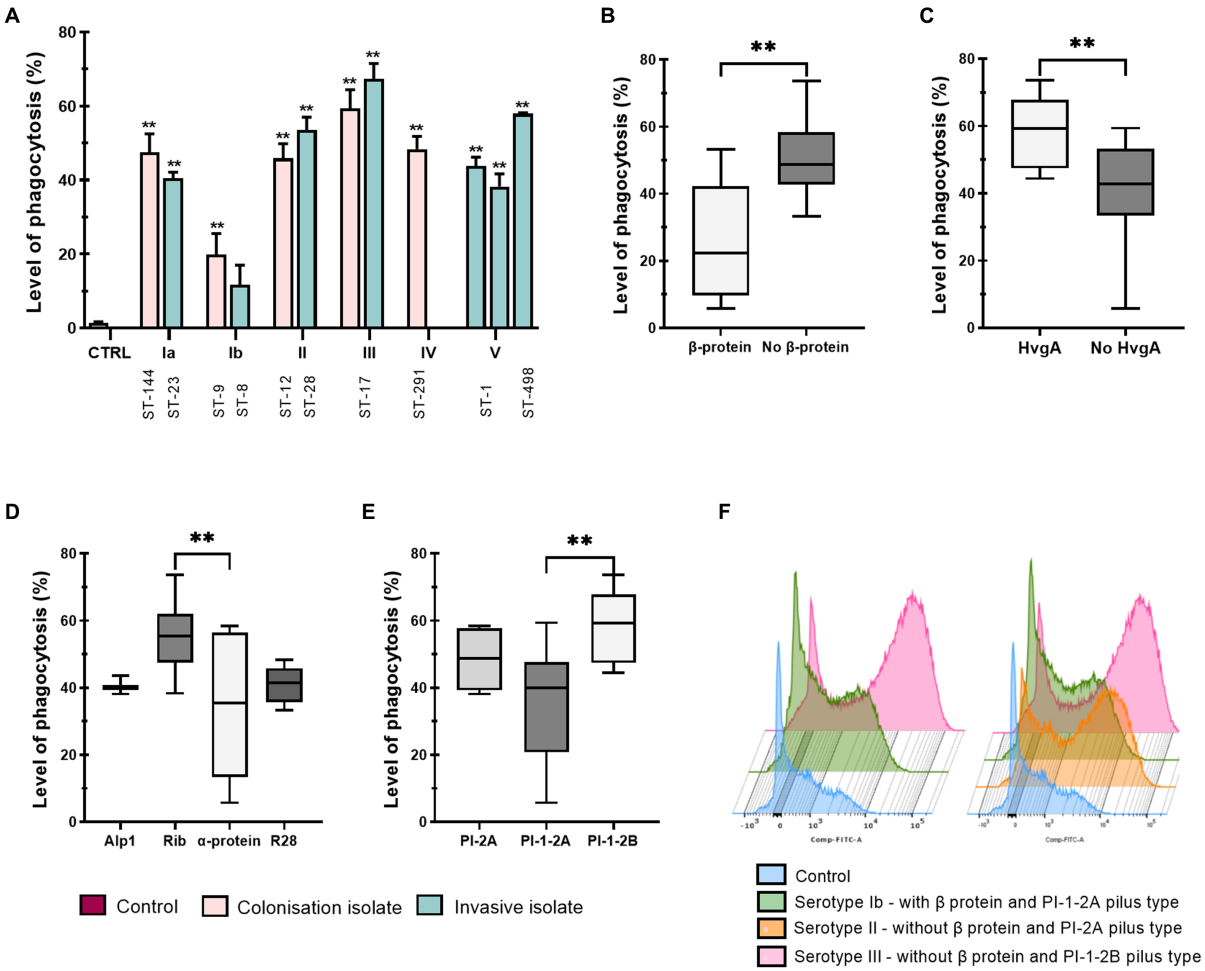
Phagocytic uptake of 12 various GBS isolates by THP-1 macrophages. Level of phagocytosis (%) after a 3h infection time at a MOI of 10:1 was measured by flow cytometry. The percentage of cells that phagocytosed the FITC-labeled GBS was determined based on the negative control histogram. **(A)** The level of phagocytic uptake (%) of GBS isolates according to serotypes and STs. The mean ± standard error (SEM) is shown for three independent biological replicates (*n*  = 3). **p  ≤ 0.01 versus non-stimulated control macrophages as determined by ANOVA with Šidák’s *post-hoc* test. Statistical differences between individual isolates are shown in Supplementary Table S1. CTRL – negative control. **(B–E)** GBS phagocytosis after stratification of data according to virulence factors **(B)** protein β, **(C)** protein HvgA, **(D)** Alp proteins and **(E)** pilus type. **(F)** Left: overlay histograms comparing phagocytosis of isolates with (green – serotype Ib) and without protein β (pink –serotype III), and negative control (blue). Right: overlay histograms of phagocytosis according to pilus type (orange – serotype Ia, pilus type PI-2A; green – serotype Ib, pilus type PI-1-2A; pink – serotype III, pilus type PI-1-2B; blue – negative control). For the boxplots, the bottom and top borders represent the 25th and 75th percentile, the middle line represents the median and the whiskers indicate variability outside the upper and lower quartiles. The Gaussian distribution of the data was determined with the Shapiro–Wilk test. **p  ≤ 0.01 as determined by un-paired *t*-test or one-way ANOVA, followed by *post-hoc* Tukey’s multiple-comparison tests.

Phagocytic uptake of 12 different live GBS isolates was determined using an optimized flow cytometry protocol. As illustrated, macrophages stimulated with all isolates showed a statistically significant increase in uptake compared with non-stimulated control macrophages (Figure 1A). However, the results showed considerable variation between isolates belonging to different serotypes, STs and CCs. The level of phagocytosis ranged from 10% for serotype Ib to over 70% for serotype III, with significantly lower phagocytic uptake of serotype Ib isolates compared to all other serotypes. Significant differences between other isolates were also observed and are shown in supplemental material (Supplementary Table S1). Stratification of data by STs demonstrated that ST-17 isolates induced the highest phagocytosis, whereas phagocytosis of ST-8 and ST-9 isolates (serotype Ib) was the lowest (Figure 1A). A high level of phagocytosis was also observed in ST-498 (serotype V) and ST-28 (serotype II) isolates, while phagocytosis of other isolates ranged from 40 to 50%. As shown in Figure 1B, the ability to phagocytose also depends on the presence of the virulence factor protein β, which is present in isolates of serotype Ib and in colonizing isolate of serotype II. However, phagocytic uptake was 20–30% higher in latter than in serotype Ib isolates. In addition, the extent of phagocytosis is also dependent on the virulence factor HvgA (Figure 1C), type of Alp proteins (Figure 1D) and pilus type (Figure 1E). Phagocytosis was the highest in isolates of serotype III, ST-17, which have the protein HvgA together with the pilus type PI-1-2B and Rib proteins. Overall, phagocytosis was significantly lower in isolates with pilus type PI-1-2A compared to pilus type PI-1-2B. However, phagocytosis capacity is independent of whether the isolate is colonizing or invasive (Supplementary Figure S5A) or of the site of infection (Supplementary Figure S5B). Phagocytosis appears to be more dependent on serotype, ST, and certain virulence factors. These data therefore suggest that different GBS isolates vary in their ability to be phagocytosed by THP-1 macrophages and that phagocytic uptake is isolate-specific. Comparison of phagocytosis of different GBS isolates is also illustrated by histograms (Figure 1F) showing differences in fluorescence intensity of phagocytosed bacteria for macrophages.

### 3.2. Colonizing isolates in particular induce high expression of co-stimulatory molecules

**Figure 2:**
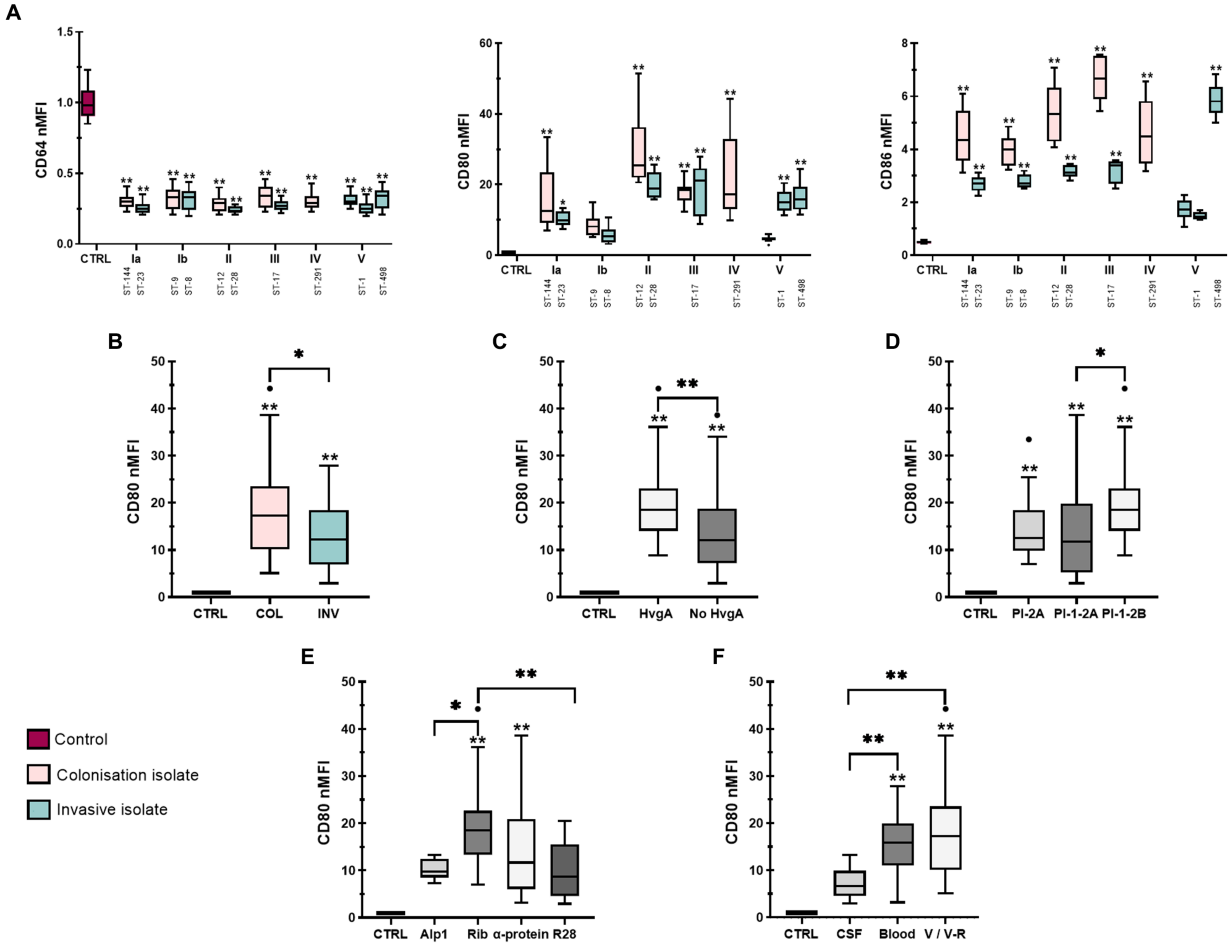
Expression of M1 macrophage phenotype markers after stimulation with 12 different GBS isolates. THP-1 macrophages were infected with GBS for 3 h (MOI 10:1). Extracellular bacteria were then removed and fresh medium with antibiotics was added. Three days later, macrophages were collected for staining and analyzed with a flow cytometer. **(A)** Plots of normalized median fluorescence intensity (nMFI) of CD64 (left), CD80 (middle) and CD86 (right). **(B–F)** Plots of nMFI of CD80 according to **(B)** clinical presentation (INV – invasive, COL – colonizing), **(C)** virulence factor HvgA, **(D)** pilus type, **(E)** Alp proteins and **(F)** specimen (CSF – cerebrospinal fluid, V/V-R – vagina/vagina-rectum, CTRL – negative control). Data from three independent biological replicates, each with three technical replicates (*n*  = 9), were normalized to negative control values. *p ≤  0.05 and **p ≤ 0.01 versus non-stimulated control macrophages (unless otherwise noted) as determined by one-way ANOVA, followed by *post-hoc* Šidák’s multiple comparisons test. Statistical differences between individual isolates are shown in Supplementary Tables S2A–C.

In addition to other functions, macrophages express specific surface molecules through which they stimulate other immune cells (Forrester et al., 2018). Therefore, the normalized median fluorescence intensity (nMFI) of six surface molecules, CD64, CD80 and CD86, characteristic of M1 phenotype and CD68, CD163 and CD206, characteristic of M2 phenotype was assessed by flow cytometry (Figures 2 3). Interestingly, stimulation with all isolates resulted in a significant decrease in the expression of the Fcγ receptor for IgG, CD64, compared with non-stimulated control cells, with little difference between the individual isolates (Figure 2A, left). Stratification of data by clinical presentation, pilus type, specimen type, or presence or absence of the virulence factors HvgA and protein β revealed no differences between isolates (Supplementary Figure S6). On the other hand, infection with all GBS isolates significantly increased the expression of macrophage co-stimulatory molecules, CD80 and CD86 (Figure 2A, middle and right). Stratification of data by clinical presentation revealed that colonizing isolates induced higher expression of co-stimulatory molecules than invasive isolates (Figure 2B and Supplementary Figure S6A). CD86 expression was most strongly induced by colonizing isolate of serotype III (ST-17), followed by colonizing isolates of serotype II (ST-12), IV (ST-291), Ia (ST-144), and Ib (ST-9) (Figure 2A, right). Conversely, CD80 expression was highest in macrophages stimulated with a colonizing isolate of serotype II (ST-12), followed by isolates of serotype III (ST-17) and IV (ST-291) (Figure 2A, middle). Consistent with phagocytic uptake, isolates of serotype Ib (ST-8 and ST-9) induced the lowest expression of CD80, whereas the expression of CD86 was the lowest in macrophages stimulated with isolates of ST-1 (serotype V). Increased expression of CD80 and CD86 was observed with isolates containing virulence factor protein HvgA (Figure 2C and Supplementary Figure S6B), pilus type PI-1-2B (Figure 2D and Supplementary Figure S6C), and Rib proteins (Figure 2E and Supplementary Figure S6D). These isolates belong to serotypes III and IV. After stratification of data by specimen, the highest CD80 and CD86 expression was observed with colonizing isolates from vagina/vagina-rectum (V/V-R), followed by isolates from blood and cerebrospinal fluid (CSF) (Figure 2F and Supplementary Figure S6E). Statistical differences between isolates are shown in Supplementary Tables S2A–C.

**Figure 3:**
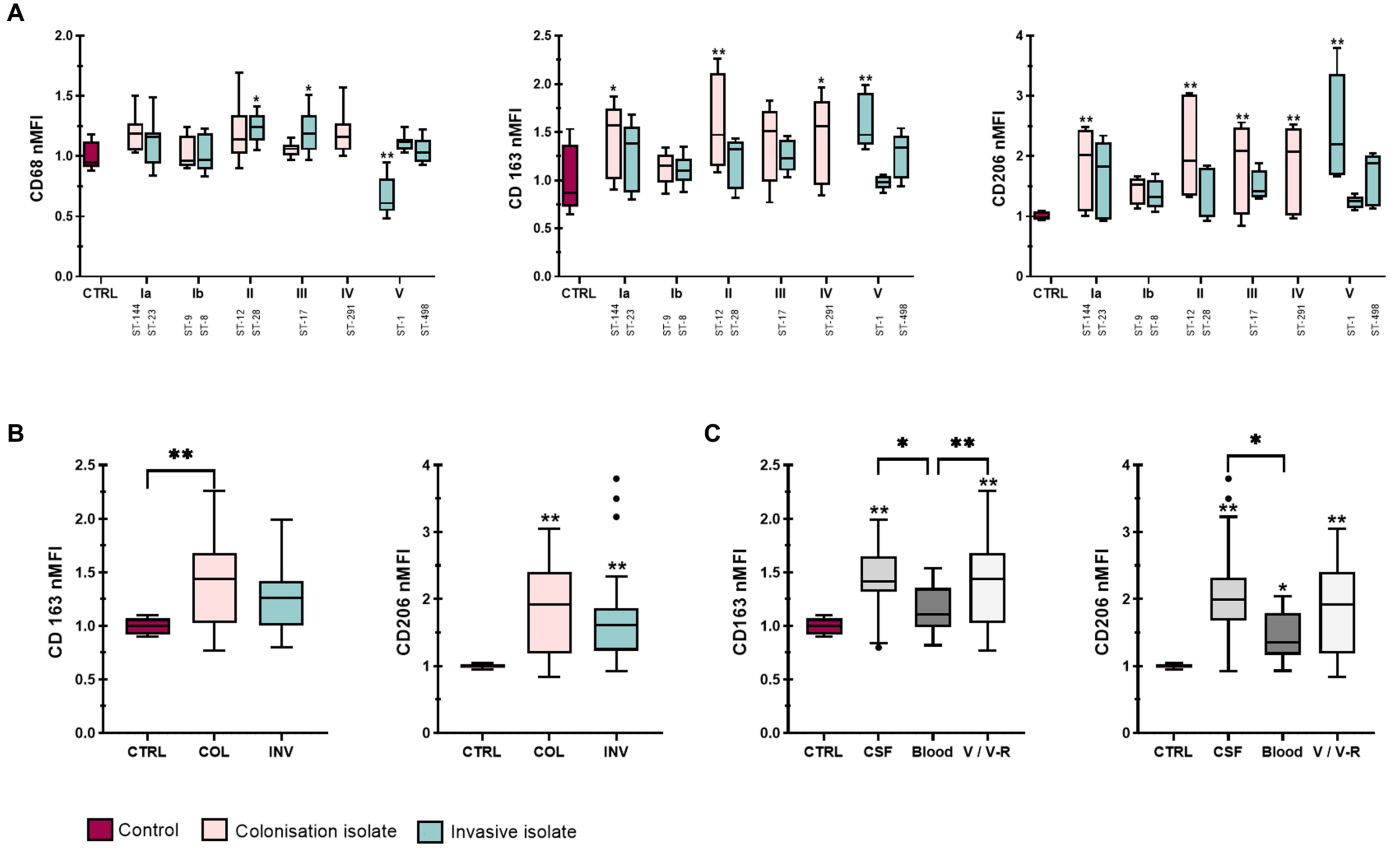
Expression of M2 macrophage phenotype markers after stimulation with 12 different GBS isolates. **(A)** Plots of normalized median fluorescence intensity (nMFI) of CD68 (left), CD163 (middle) and CD206 (right) on THP-1 macrophages, stimulated with GBS isolates of different serotypes and STs. **(B,C)** Plots of nMFI of CD163 and CD206 expression after stratification of data according to clinical presentation (INV – invasive, COL – colonizing) and specimen (CSF – cerebrospinal fluid, V/V-R – vagina/vagina-rectum, CTRL – negative control). Data from three independent biological replicates, each with three technical replicates, are shown (*n*  = 9). *p  ≤ 0.05 and **p  ≤  0.01 versus non-stimulated control macrophages (unless otherwise noted) as determined by Kruskal-Wallis test with Dunn’s *post-hoc* test. Statistical differences between individual isolates are shown in Supplementary Tables S2D–F.

### 3.3. M2 markers are most highly expressed in macrophages infected with isolates from CSF

CD68, CD163, and CD206 expression was less variable between isolates (Figure 3A). While there were no statistically significant differences in CD68 expression between colonizing and invasive isolates or non-stimulated control (Supplementary Figure S6A), the data showed higher CD68 expression for isolates with the virulence factor HvgA compared with both isolates without HvgA and control macrophages (Supplementary Figure S6B). Interestingly, the lowest CD68 expression was observed for isolate 6 (serotype V, ST-1) obtained from CSF (Figure 3A, left). In addition, the data showed slightly higher expression of CD163 for colonizing isolates (Figure 3B) from V/V-R (Figure 3C). The expression of CD163 and CD206 was also statistically higher for isolates from V/V-R and CSF compared with control, as well as for isolates from CSF compared with isolates from blood. A statistical difference was also observed in CD163 expression between isolates from V/V-R and isolates from blood (Figure 3C). Comparisons of other virulence factors for M2 phenotype markers were statistically insignificant. Differences between individual isolates are shown in Supplementary Tables S2D–F.

### 3.4. Colonizing isolates are significantly more cytotoxic compared to invasive isolates

**Figure 4:**
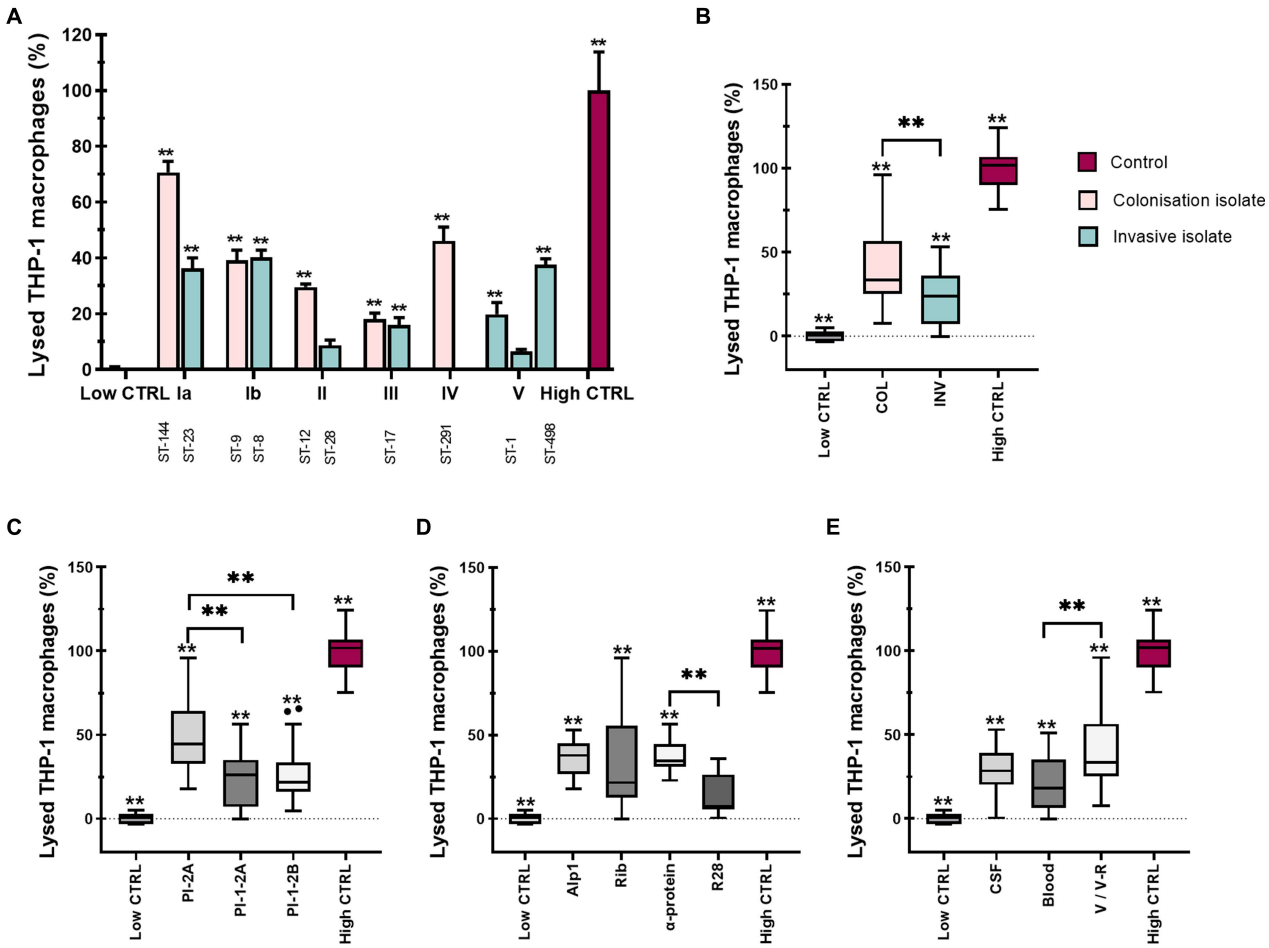
Various GBS isolates are differentially cytotoxic to THP-1 monocyte-derived macrophages. THP-1 macrophages were stimulated with 12 different GBS isolates at a MOI of 10 for 3 h, after which supernatants were collected and cytotoxicity was measured by LDH release. **(A)** Cytotoxicity of GBS isolates according to serotypes and STs. Data from three independent biological replicates, each with three technical replicates, are shown as mean ± SEM (*n* = 9). **p ≤ 0.01 versus low control as determined by one-way ANOVA and *post-hoc* Šidák’s test. All isolates were statistically significant with the high control. Statistical differences between individual isolates are shown in Supplementary Table S3A. **(B–E)** GBS-mediated cell cytotoxicity according to **(B)** clinical presentation (COL – colonizing, INV – invasive), **(C)** pilus type, and **(D)** Alp proteins and **(E)** specimen (CSF – cerebrospinal fluid, V/V-R – vagina/vagina-rectum). **p  ≤ 0.01, as determined by un-paired *t*-test or Kruskal-Wallis test, followed by *post hoc* Dunn’s multiple-comparison test, respectively.

Since several studies have reported that GBS isolates damage various eukaryotic cell types, including macrophages, we quantified the cytotoxic effect of different GBS isolates on macrophages by measuring the amount of LDH released in the culture supernatants. Consistent with phagocytosis and macrophage markers expression, we hypothesized that there would also be statistically significant differences in GBS cytotoxicity between isolates. This hypothesis was confirmed by colonizing isolate of serotype Ia (ST-144) inducing the highest (70%) macrophage lysis, followed by the colonizing isolate of serotype IV (ST-291, 50%), isolates of serotype Ib (ST-8 and ST-9, 40%) and invasive isolate of serotype Ia (ST-23, 35%) (Figure 4A). Statistical differences between isolates are shown in Supplementary Table S3A. When the data were pooled according to clinical presentation, colonizing isolates caused significantly higher macrophage lysis compared with invasive isolates (Figure 4B). The highest lysis was caused by isolates from V/V-R, followed by isolates from CSF and finally isolates from blood (Figure 4E). Stratification of data by pilus type revealed statistically significant differences between pilus type PI-2A compared with pilus type PI-1-2A and PI-1-2B, respectively (Figure 4C), whereas stratification by Alp proteins showed the highest cytotoxicity for isolates, possessing Alp1 and α-protein (Figure 4D). Similar results were obtained when macrophages were stimulated with GBS at a MOI of 20:1 (Supplementary Figure S7 and Supplementary Table S3B).

### 3.5. GBS infection enhances both glycolytic and oxidative metabolism in an isolate specific manner

**Figure 5:**
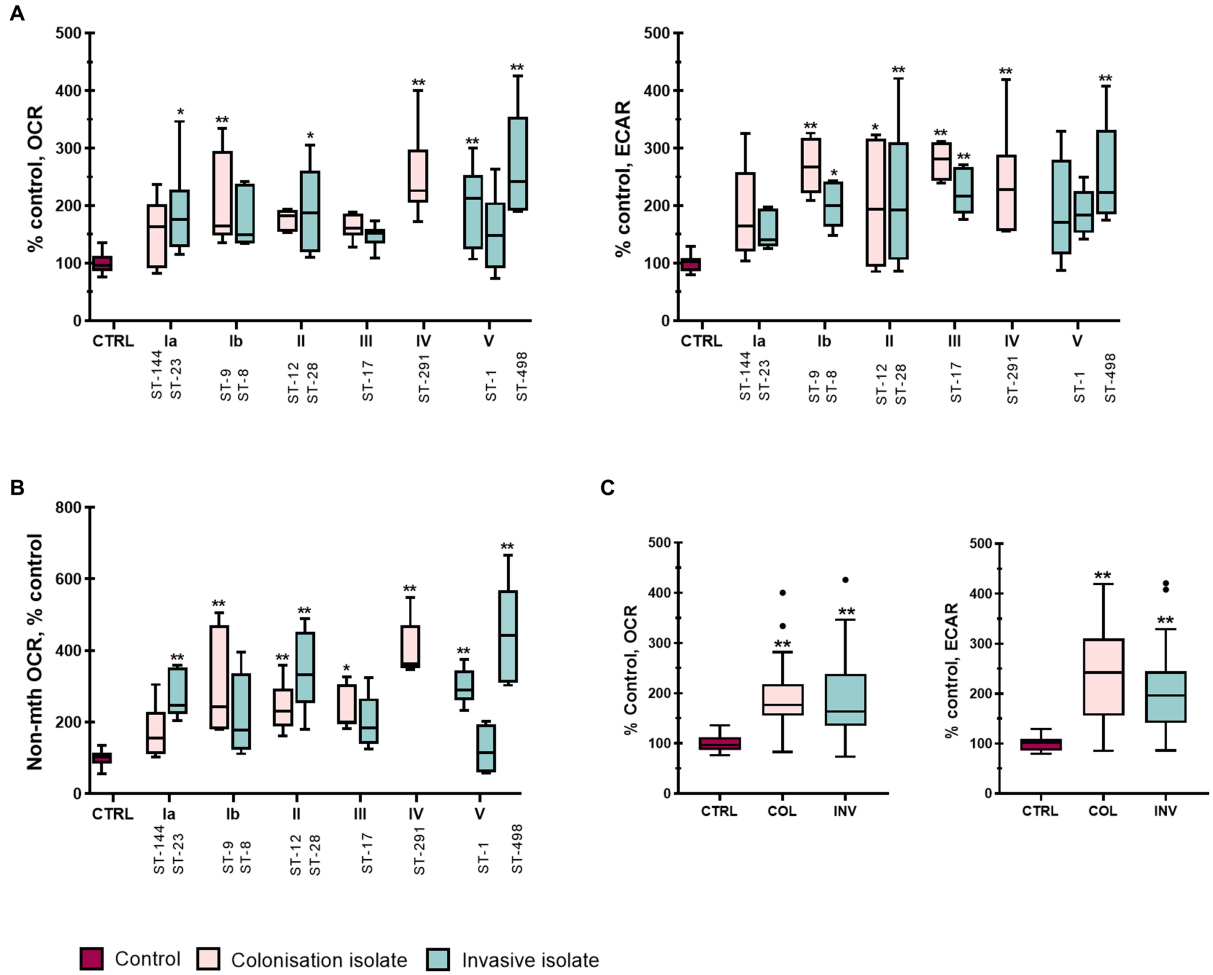
GBS isolates enhance overall metabolism of stimulated THP-1 macrophages. Basal oxygen consumption rate (OCR) and extracellular acidification rate (ECAR) were analyzed in real-time 4 h post-infection using a Seahorse XFe24 Extracellular Flux analyzer and a modified Real-Time ATP Production Rate Assay. Ten consecutive measurements cycles were performed to detect any changes in metabolism, with measurement number 11 considered the basal OCR and ECAR. Two independent experiments, each with three technical replicates, were performed (*n* = 6). **(A)** OCR (left) and ECAR (right) of THP-1 macrophages stimulated with GBS isolates of different serotypes and STs. **(B)** Non-mitochondrial oxygen consumption (non-mth OCR) was measured after injection of rotenone and antimycin-A and normalized to control. **(C)** Metabolic phenotype (basal OCR and ECAR) induced by isolates according to their clinical presentation. *p  ≤ 0.05 and **p  ≤ 0.01 versus non-stimulated control macrophages as determined by ANOVA with Šidák’s *post-hoc* test or Kruskal-Wallis with Dunn’s *post-hoc* test. Statistical differences between individual isolates for basal and non-mitochondrial OCR are shown in Supplementary Tables S4, S8.

Because macrophage functions depend on their activation, we next examined how infection with different GBS isolates modulates the metabolic profile of THP-1 macrophages by measuring oxygen consumption rate (OCR) and extracellular acidification rate (ECAR) as indicators of cellular respiration and glycolysis, respectively. Stimulation with GBS isolates increased both glycolysis and cellular respiration compared with non-stimulated control macrophages (Figure 5A). Six of 12 isolates significantly increased OCR, whereas eight of 12 isolates significantly increased ECAR compared with control, with a more pronounced increase in ECAR. Statistical differences were also observed between individual isolates, especially in OCR (Supplementary Table S4). The lowest OCR (about 150% of control) was observed with serotype III (ST-17), followed by serotype Ia (ST-144, ST-23) and II (ST-12, ST-28). On the other hand, isolates of serotype III (ST-17) caused the highest increase in ECAR (250 to over 300% of control). For other isolates, the differences were not as clear, but they increased both ECAR and OCR compared with control. Stratification of data by clinical presentation (Figure 5C) revealed no difference between colonizing and invasive isolates, although both showed significant differences from control cells in OCR and ECAR, with more pronounced changes in the latter. Comparison of other virulence factors also revealed no statistically significant differences.

To further assess the metabolic shift induced by GBS stimulation, the ratios of ECAR to OCR were compared for individual isolates (Supplementary Figures S9A, S10 and Supplementary Table S7). The ECAR/OCR ratio was significantly increased compared with control only by colonizing isolate of serotype Ib and isolates of serotype III, which had the highest ECAR/OCR ratio. On the other hand, the ECAR/OCR ratio was the lowest in isolates of ST-291, ST-1, and ST-498 and was practically unchanged compared with control (Supplementary Figures S9A, S10A,E,F). ECAR/OCR ratios for all isolates within each serotype and compared with control are also shown in Supplementary Figure S9C (for isolates of serotype III, ST-17) and S10. Overall, the results showed considerable variability in the ability of each isolate to induce either oxidative or glycolytic metabolism or both in THP-1 macrophages, with no clear difference between colonizing and invasive isolates.

### 3.6. Following infection, ATP is produced mainly by aerobic glycolysis

**Figure 6:**
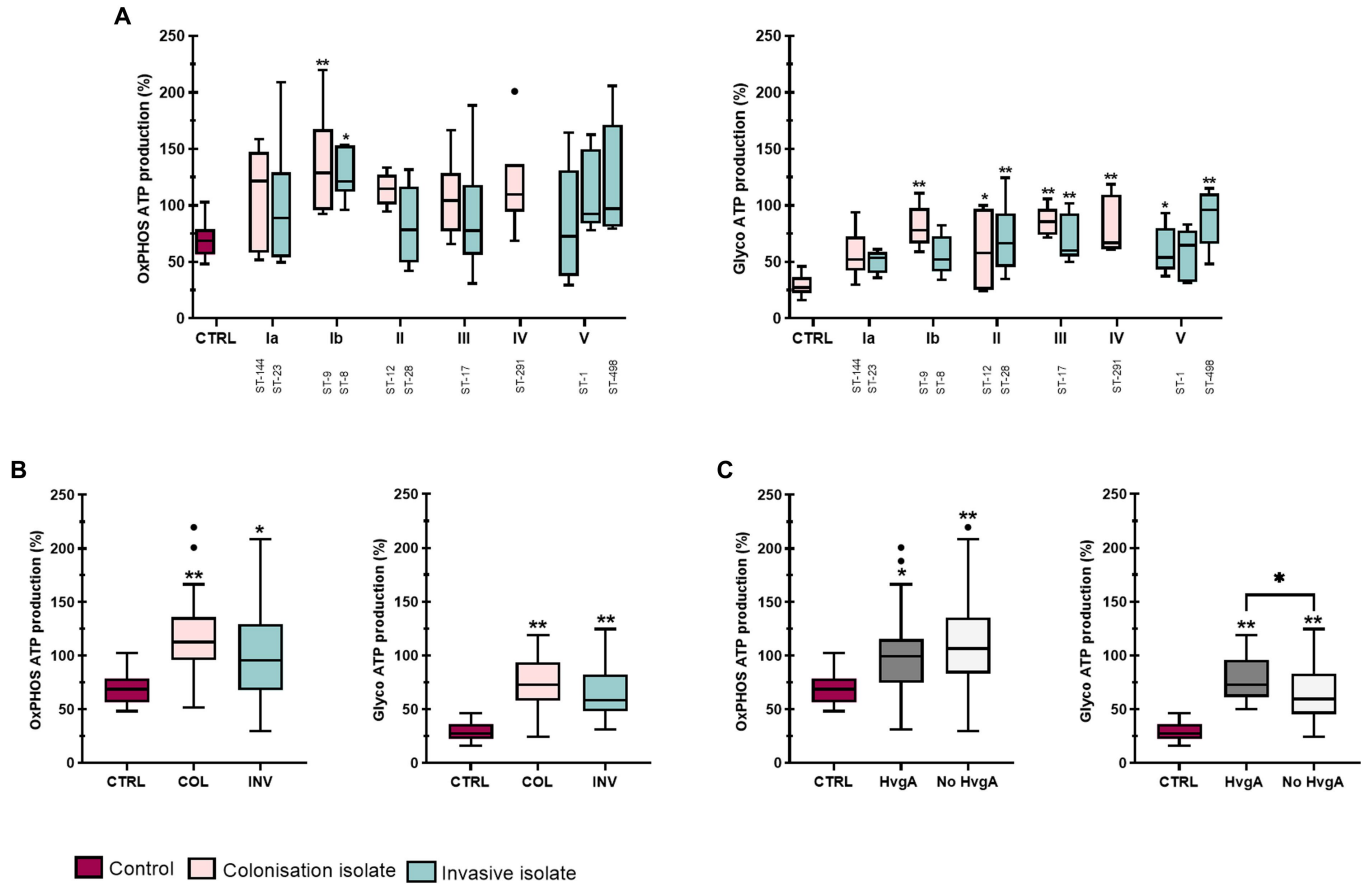
ATP-production in THP-1 macrophages after stimulation with 12 different GBS isolates. ATP production was determined 4 h after infection with live bacteria using a modified Seahorse Real Time ATP Production Rate Assay. Baseline OCR and ECAR were obtained from the 11th measurement cycle under basal conditions. OCRs after injections of oligomycin and rotenone plus antimycin-A were used to calculate ATP production from OxPhos and glycolysis according to manufacturer’s instructions. Data from two independent experiments, each with three technical replicates, were obtained (*n*  = 6). **(A)** Boxplots show ATP production rate (% total ATP production in control) from OxPhos (left) or glycolysis (right) after stimulation with GBS isolates belonging to different serotypes and STs. **(B,C)** ATP production rate (% total ATP production in control) in THP-1 macrophages according to **(B)** clinical presentation (colonizing/invasive) and **(C)** presence of virulence factor HvgA. *p  ≤ 0.05 and **p  ≤ 0.01 versus non-stimulated control macrophages as determined by ANOVA with Šidák’s *post-hoc* test or Kruskal-Wallis with Dunn’s *post-hoc* test. Statistical differences between individual isolates are shown in Supplementary Table S5.

To quantify more precisely the effect of GBS stimulation on glycolysis and oxidative phosphorylation (OxPhos), we also measured OCR and ECAR in response to olygomycin and antimycin + rotenone-A injections and calculated ATP production from both processes according to the Seahorse Real-Time ATP Rate Assay. GBS infection increased ATP production rate in THP-1 macrophages, particularly glycolytic ATP production rate, compared with non-stimulated control macrophages (Figure 6A and Supplementary Table S5). Except for isolates of ST-144 and ST-8, all GBS isolates significantly increased glycolytic ATP production compared with control, whereas OxPhos ATP production was significantly increased only in isolates of serotype Ib. This is consistent with the above observation that GBS infection significantly increases ECAR in particular. After stratification of data according to clinical presentation (Figure 6B), both colonizing and invasive isolates induced significantly increased ATP production from glycolysis and OxPhos compared with control macrophages, with colonizing isolates showing a statistically non-significant trend toward higher ATP production compared with invasive isolates. However, pooling of data according to the presence or absence of the virulence factor protein HvgA in isolates of serotype III (ST-17) and serotype IV (ST-291) revealed that stimulation with HvgA isolates induced statistically higher glycolytic (but not OxPhos) ATP production compared with isolates lacking protein HvgA (Figure 6C). However, comparisons of other virulence factors were insignificant. Comparison of the ratio between the glycolytic and OxPhos ATP production rates revealed that the ratio was the highest for isolates of serotype III, indicating the largest relative shift of ATP production toward glycolysis (Supplementary Figures S9B,D and Supplementary Table S6). On the other hand, the ratio was the lowest in isolates of serotype Ib and practically unchanged compared with the control, reflecting the high rate of OxPhos ATP production observed for this serotype. For isolates of serotype III (ST-17), both colonizing and invasive isolates significantly increased the ratio of glycolytic to OxPhos ATP production compared to control, whereas no significance was observed between individual isolates of serotype III (Supplementary Figure S9D). However, the trend showed a higher ratio of glycolytic to OxPhos ATP production for invasive isolates, in contrast to the ECAR/OCR ratio (Supplementary Figures S9C,D). The ratios of glycolysis to OxPhos ATP production for other isolates within each serotype are shown in Supplementary Figure S10.

### 3.7. Non-mitochondrial oxygen consumption

The discrepancy between the ratios of ECAR/OCR and glycolytic/OxPhos ATP production for serotype III mentioned above and the smaller magnitude of changes in OxPhos ATP production compared with changes in basal OCR prompted us to also examine the non-mitochondrial oxygen consumption rate, i.e., OCR insensitive to rotenone and antimycin-A (Figure 5B and Supplementary Table S8). We found that non-mitochondrial OCR was substantially and significantly increased for all GBS isolates except the colonizing isolates of serotype Ia and the invasive isolates of serotype Ib and III and one of the invasive isolates of serotype V (Figure 5B). This increase largely paralleled the increase in basal OCR (Figure 5A), suggesting that the latter was caused in large part by increased non-mitochondrial oxygen consumption, possibly by NADPH oxidase. Overall, the data indicates that most of the studied GBS isolates induce a substantial upregulation of glycolytic ATP production, while the increase in oxygen consumption is predominately from non-mitochondrial sources such as NADPH oxidase. Although no significant overall difference between colonizing and invasive isolates could be observed, important differences exist in the metabolic response of macrophages to different GBS isolates.

### 3.8. Macrophages stimulated with different GBS isolates show different morphology

**Figure 7:**
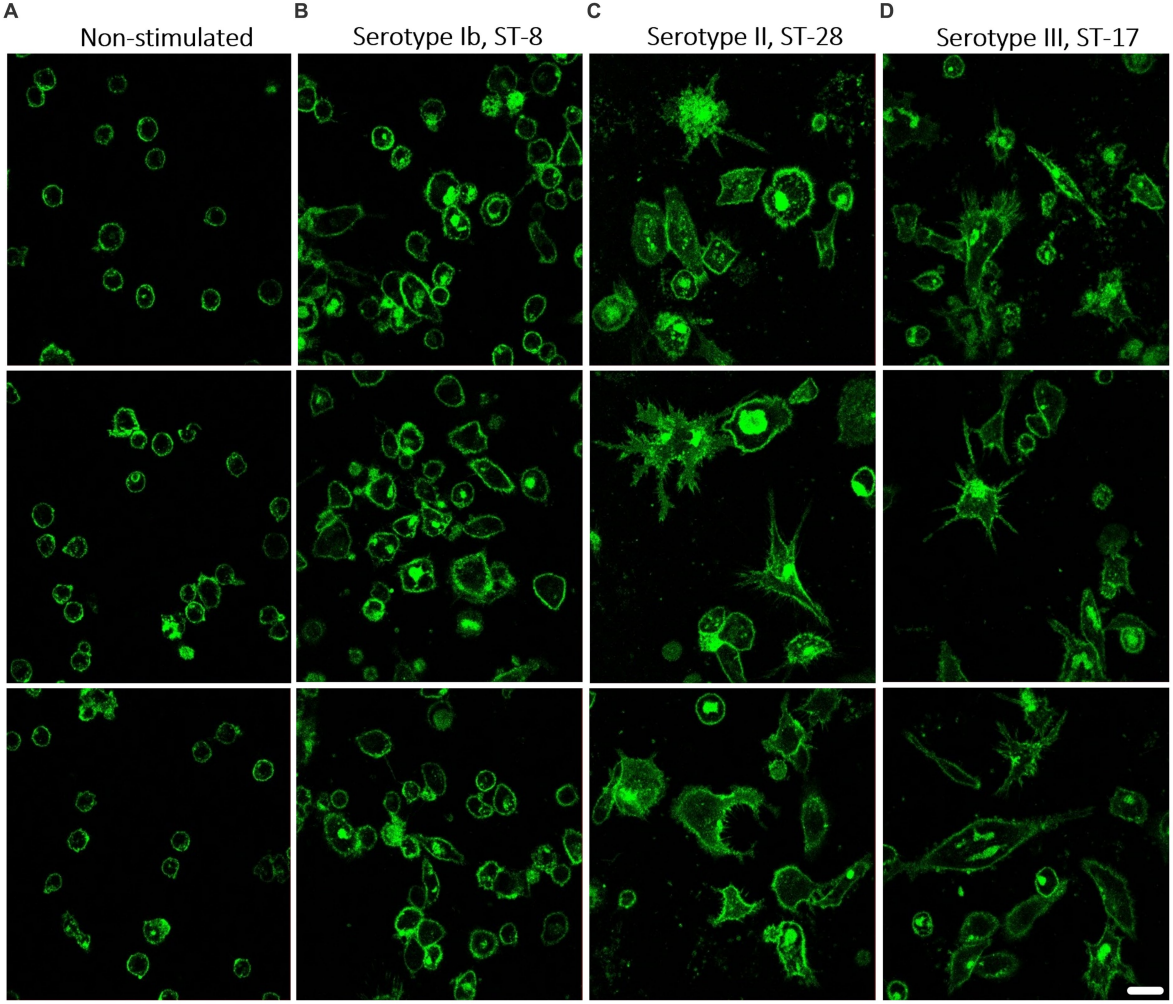
The morphology of THP-1 macrophages after stimulation with different GBS isolates. Macrophages were stimulated with GBS isolates belonging to 3 different serotypes and STs. After incubation, the bacteria were removed and fresh medium with antibiotics was added for another 3 days. Macrophages were then stained with CellMask Green Plasma Membrane Stain and visualized with a confocal microscope. Representative images of three technical replicates for each isolate are shown. **(A)** Non-stimulated THP-1 macrophages as a negative control. Morphology of THP-1 macrophages stimulated with **(B)** invasive isolate of serotype Ib, ST-8, with the virulence factor protein β, **(C)** invasive isolate of serotype II, ST-28, and **(D)** invasive isolate of serotype III, ST-17. Cell membranes are stained green, 600x magnification. Scale bar: 10 μm.

To determine whether stimulated macrophages also differ morphologically, the membranes of macrophages stimulated with three different isolates were stained with CellMask dye and observed under a fluorescence microscope. Based on previous results, we chose one isolate that elicits a weak immune response (isolate 203, serotype Ib, ST-8) and two isolates that elicit a stronger response (isolate 211, serotype II, ST-28 and isolate 231, serotype III, ST-17). Non-stimulated control macrophages showed a relatively round shape, whereas macrophages stimulated with GBS isolates became flatter, more elongated, and branched (Figure 7). It can also be seen that macrophages stimulated with isolate of serotype Ib, ST-8 (Figure 7B) were significantly less morphologically branched than macrophages stimulated with isolates of serotype II, ST-28 (Figure 7C) and serotype III, ST-17 (Figure 7D). During the 3-day incubation period, the latter developed a number of pseudopodia with which they sense and engulf bacteria. Quantification of morphological changes, in which morphological changes were divided into three categories (MC1 – unbranched, round; MC2 – elongated, but without pseudopodia; MC3 – highly differentiated, numerous pseudopodia), also showed that isolates of serotype Ib induced significantly less extensive morphological changes than isolates of serotypes II and III (Supplementary Figure S11). These observations are consistent with results obtained by other methods, suggesting that different GBS isolates differ in the strength of the induced immune response.

### 3.9. Real-time microscopy of macrophage interaction with GBS isolates

To capture real-time changes in macrophage interactions with GBS isolates, three different GBS isolates were selected: isolate 231 (serotype III, ST-17), which showed the highest phagocytic uptake and a macrophage shift toward glycolysis, isolate 9427 (serotype Ia, ST-144), which showed the highest cytotoxicity, and isolate 203 (serotype Ib, ST-8), which showed the lowest macrophage response. During a 3 h incubation with bacteria, macrophages stimulated with all three isolates became strongly activated compared with non-stimulated, control macrophages (Supplementary Videos S1–S4). While real-time microscopy showed no visible differences between isolates of serotype Ib (Supplementary Video S3) and III (Supplementary Video S4), a higher number of swollen and ruptured, probably dead macrophages was observed with isolates of serotype Ia (Supplementary Video S2). In addition, macrophages stimulated with all isolates differentiated significantly more than non-stimulated control macrophages. They also began to migrate around the surface, branching, sensing and engulfing both bacteria and other infected macrophages.

## 4. Discussion

Invasive GBS infections pose a serious threat not only to newborns but also to elderly and immunocompromised individuals. Due to increasing antibiotic resistance or therapy ineffectiveness, the development of new, alternative therapies is needed. Within 10 serotypes, GBS isolates are further classified into a number of ST and CC types, which differ in capsular polysaccharides and possess various virulence factors, thus triggering a different immune response (Flaherty et al., 2021).

In the present study, a THP-1 cell line was used to study the innate immune response of macrophages to 12 distinct GBS isolates. Monocytes were differentiated into macrophages by incubation with 100 nM PMA for 72 h, followed by a 5-day rest in medium without PMA. The differentiation protocol was chosen based on preliminary results where duration of PMA incubation, concentration and rest period were varied (Supplementary Figures S1–S4). The selected protocol yielded the highest viability of macrophages with adequate expression of differentiation markers. Additionally, the 3-day PMA and 5-day rest protocol was also validated in a study by Daigneault et al. (2010).

Using an optimized phagocytosis protocol on a flow cytometer, we demonstrated that diverse GBS isolates are phagocytosed differently. Differences were observed both between individual serotypes and between isolates of the same serotype but of a different ST type. However, isolates within the same serotype were mostly phagocytosed similarly, as statistical differences within individual serotypes were only obtained for isolates of serotype V, belonging to different STs. While Rogers et al. (2018) first reported that serotype, ST type and invasiveness of isolates did not affect phagocytosis, our data suggest otherwise. This could likely be because they used heat inactivated GBS isolates whereas we examined the influence of live, opsonized bacteria. Our results are also in agreement with previous studies demonstrating isolate-specific differences in phagocytic uptake of GBS (Korir et al., 2017a; Flaherty et al., 2021).

The phagocytic uptake was the highest for ST-17 isolates (serotype III) (70%), consistent with the observations by other studies (Cornacchione et al., 1998; Korir et al., 2017a), whereas for ST-8 and ST-9 isolates (serotype Ib) the uptake was only around 10–20%. Previous genotyping of GBS isolates used in our study (Perme et al., 2020) has shown that isolates of serotype Ib (ST-8 and ST-9) possess the virulence factor protein β, which prevents the deposition of C3b component of the complement on the bacterial surface, thereby reducing opsonophagocytosis (Korir et al., 2017b). In addition, it also binds to the Siglec-5 surface receptor, further inhibiting phagocytosis (Patras and Nizet, 2018). Since bacteria were opsonized in human serum as a source of complement prior to stimulations, the presence of protein β could explain the low phagocytic uptake for isolates of serotype Ib. The protein β is also expressed by a colonization isolate of ST-12. However, the latter belongs to serotype II and has different capsular polysaccharides and different amount of sialic acid, which are two factors directly involved in immune system. Nevertheless, both our and other studies demonstrate that hypervirulent isolates of ST-17 (serotype III) have the highest ability to be phagocytosed by macrophages, regardless of whether the isolates were pre-opsonized, whereas differences between the other isolates confirm that serotype indeed affects the ability of GBS to be phagocytosed.

Our data further demonstrate that phagocytosis is also affected by the pilus type. The importance of pili in the virulence of GBS has been investigated and three types of pili (PI-2A, PI-1-2A, and PI-1-2B) have been described (Landwehr-Kenzel and Henneke, 2014). Isolates contain at least one or a combination of two types of pili. PI-1 pili are important for evading phagocytosis by macrophages but are not involved in adhesion to the host cell (Jiang et al., 2012). PI-2A pili are important for biofilm formation, whereas PI-2B pili increase intracellular survival in macrophages (Chattopadhyay et al., 2011) and contribute to the invasiveness of GBS (Lazzarin et al., 2017).

Studies have also shown that isolates possessing PI-1-2B pilus type are phagocytosed to a higher extent compared to the isolates possessing other type of pili (Lazzarin et al., 2017). The latter express the protein Spb1, which increases the adhesiveness, enhances phagocytosis and at the same time enables intracellular survival of GBS, contributing to higher virulence of GBS (Chattopadhyay et al., 2011). Of the isolates used in our study, the above pilus type is present in hypervirulent isolates of ST-17 (serotype III) and a colonizing isolate of serotype IV, which were phagocytosed more efficiently than isolates possessing other types of pili. The aforementioned isolates of serotype III and IV also possess the adhesin HvgA, which facilitates binding of the bacterium to the host cell, which may be another reason for the enhanced phagocytosis of these isolates.

Moreover, surface immunogenic adhesins Rib, R28, Alp1 and α-proteins, belonging to the Alpha like protein family, are important for GBS virulence (Pietrocola et al., 2018). The most immunogenic proteins are the Rib proteins (Bianchi-Jassir et al., 2020) expressed by isolates of serotype III and IV, for which we observed the highest phagocytic uptake. Our results suggest that in addition to the antibody response, the Rib proteins as reported so far, also contribute to the increased phagocytosis and are importantly involved in immunogenicity (Pawlowski et al., 2022).

Further, colonizing isolates from V/V-R showed significantly higher lysis of macrophages compared to invasive isolates from blood with the highest percentage (70%) of lysis with a colonizing isolate of serotype Ia (ST-144), followed by the colonizing isolate of serotype IV (ST-291). Studies have shown that the GBS toxin β-hemolysin/cytolysin is capable of inducing pyroptosis in macrophages (Whidbey et al., 2015). It was demonstrated that in addition to pyroptosis, GBS is also capable of inducing macrophage apoptosis (Fettucciari et al., 2000). Real-time microscopy also showed that macrophages swelled and ruptured after stimulation with colonizing isolates of serotype Ia (ST-144), whereas this was not observed with isolates of serotype Ib (ST-8) and especially serotype III (ST-17). However, further studies are needed to clarify which type of cell death occurs.

We also observed that more cytotoxic isolates induced a larger hemolytic zone on blood agar and multiplied faster in co-culture than others, which is in agreement with a study by Sendi et al. (2009) showing that highly hemolytic isolates grew markedly faster compared to the less hemolytic ones. Our cytotoxicity results indicate that colonizing isolates are more cytotoxic but less invasive due to macrophage lysis, which releases them into the environment where they are exposed to further immune response or antibiotics. In contrast, less cytotoxic isolates seem to be more invasive as they do not kill host macrophages but rather use them to survive by evading other immune cells and/or antibiotics, and also facilitate dissemination throughout the body. A similar, Trojan horse phenomenon (Mittal et al., 2010; Sagar et al., 2013; Gendrin et al., 2018) has been already described for other pathogen bacteria, suggesting that bacteria can survive within macrophages and even use them to travel intracellularly in the circulation (Segura et al., 1998; Laskay et al., 2003; Thwaites and Gant, 2011). In line with these observations, our data show that invasive isolates of serotype III, II and V are the least cytotoxic but at the same time are more invasive as they were obtained from blood or CSF, confirming our hypothesis that invasive isolates, at least in the initial stages of infection, exploit macrophages to evade the immune system and survive in the host. Our results also showed the highest cytotoxicity for isolates possessing PI -2A-type of pili and Alp1 and α-proteins, but further investigation is needed to understand the correlation between the above factors and cytotoxicity.

Macrophages are extremely plastic cells, capable of switching the phenotype depending on the stimuli and signals from the microenvironment (Shapouri-Moghaddam et al., 2018). In response to pathogens, they express specific surface molecules through which they regulate and dictate further immune responses (Forrester et al., 2018). While inflammatory macrophages are defined by higher expression of co-stimulatory molecules CD80 and CD86, immunosuppressive macrophages express higher levels of scavenger receptor CD163 and mannose receptor CD206 (Takiguchi et al., 2021). Surprisingly, our data demonstrate that 3-day stimulation with all GBS isolates statistically decreased the expression of Fcγ receptor, CD64. The latter recognizes the Fc region of IgG and therefore plays a central role in linking the cellular and humoral arms of the immune response (Mittal et al., 2010). On the contrary, studies on neutrophils and monocytes have shown that CD64 expression is increased during septicemia and even proposed it as a diagnostic marker for early-onset neonatal infections (Ng et al., 2004; Groselj-Grenc et al., 2008). A possible explanation could be that GBS non-specifically interacts with CD64 and uses it to invade macrophages, while simultaneously internalizing the receptor, thereby decreasing its surface expression. Namely, a study in *E. coli* showed that OmpA protein interacts with the FcγRIa receptor, allowing the bacteria to bind to and invade macrophages (Mittal et al., 2010). Nonetheless, more studies investigating the interaction of GBS with CD64 are needed to confirm this hypothesis.

Furthermore, all GBS isolates significantly increased the expression of CD80 and CD86. The latter are expressed on the surface of antigen presenting cells (APC) in response to pathogens (Sansom et al., 2003). While CD80 expression was similar among isolates within the same serotype, CD86 expression varied widely depending on whether the isolate was colonizing or invasive. Interestingly, particularly CD86 was significantly increased, especially for colonizing isolates. Since CD80 and CD86 are required for further T cell activation (Sansom et al., 2003), increased expression would also likely result in enhanced T cell activation and more rapid clearance of infection *in vivo*. Invasive isolates, on the other hand, limit further immune responses by inducing less co-stimulatory molecules, thereby increasing their survival in the host. Moreover, *in vitro* and *in vivo* studies have shown that increased expression of CD80 and concomitant decreased expression of CD86 are associated with a more severe infection and inflammation, whereas increased expression of CD86 in septic patients is thought to have a protective function (Nolan et al., 2009). Although *in vitro* conditions differ from those *in vivo*, our data appear to be consistent with aforementioned studies, as invasive isolates obtained from blood (serotypes II, III) strongly induced CD80 but only weakly induced CD86 expression, while the opposite was observed in colonizing isolates. Consistent with the phagocytosis results, CD80 was least induced by isolates of serotype Ib which also induced low expression of CD86.

Interestingly, invasive isolates obtained from CSF appear to elicit the lowest immune response according to surface molecules expression. The latter (isolate 229, serotype Ia, ST-23 and especially isolate 6, serotype V, ST-1) only weakly induced CD80 and CD86 expression but, surprisingly, statistically higher expression of CD163 and CD206 compared with isolates obtained from blood. Recently, CD163 was found to function not only as a scavenger receptor for hemoglobin-haptoglobin complexes (Tippett et al., 2011) but also as an innate immune sensor for both Gram positive and Gram negative bacteria (Fabriek et al., 2009). Therefore, possible explanation could be that isolates from CSF bind less to the CD163, resulting in its apparent higher expression, as more CD163 is available to bind monoclonal antibodies, whereas invasive isolates from blood may bind more strongly to the CD163 leading to a septic state due to the more severe inflammation. Moreover, CD163 sheds from the cell membrane during severe bacterial infection (Hintz et al., 2002; Sulahian et al., 2004), which would explain lower CD163 expression by isolates from blood but not isolates from V/V-R and, interestingly, CSF. It is possible that CD163 sheds from the cell membrane at different times, depending on the bacterial isolate, which would explain its higher expression upon stimulation with isolate 6 (serotype V, ST-1).

Interestingly, macrophages stimulated with isolate 6 had the lowest expression of co-stimulatory molecules CD80 and CD86, along with CD68, i.e., a myeloid-specific surface marker, routinely used as a histochemical/cytochemical marker of inflammation (Chistiakov et al., 2017). Thus, the low expression of inflammatory markers with concomitant high expression of immunosuppressive markers could explain the ability of the mentioned isolate to progress to CSF.

Finally, as recent findings point to the importance of metabolism in shaping the functional phenotype of macrophages in response to various microbial stimuli, the metabolic assay was performed on macrophages exposed to 12 different GBS isolates. Numerous studies have already confirmed that stimulations with bacterial LPS or Th1 cytokines and Gram negative bacteria, *Mycobacterium tuberculosis*, or intracellular bacteria such as *Listeria monocytogenes,* shift macrophage metabolism toward increased glycolysis while simultaneously reducing mitochondrial respiration (so-called M1 or classically activated pro-inflammatory macrophages) (Gleeson et al., 2016; Fleetwood et al., 2017; Cumming et al., 2018; Escoll and Buchrieser, 2018; Russell et al., 2019; Wang et al., 2019). However, *in vitro* stimulations of macrophages with dead and live bacteria (both Gram negative and especially Gram positive) have shown that bacterial infection enhances both glycolysis and mitochondrial respiration (Lachmandas et al., 2016). Since no study has been conducted with *S. agalactiae*, ECAR and OCR were measured as readouts for glycolysis and cellular respiration using the Seahorse Extracellular Flux Analyzer. Our data showed an increase in both glycolysis and mitochondrial respiration in stimulated macrophages, with the increase in glycolysis being more pronounced. Correspondingly, ATP production, particularly from glycolysis, also increased significantly compared with the control macrophages. Similar observations were obtained by Lachmandas et al. (2016) after stimulations with whole bacterial lysates of *S. aureus, M. tuberculosis*, and *E. coli* and with the TLR2 ligand Pam_3_CysSK_4_ (P3C). In contrast to the LPS stimulations, increase in overall metabolic activity was observed. These data therefore suggest that different stimuli that elicit an M1-like phenotype are not necessarily equivalent, necessitating individual assessment of the metabolic responses they induce.

While most studies have only examined the effects of a single bacterium on immune cell metabolism, to our knowledge we were the first to examine the effect of 12 different GBS isolates on THP-1 macrophage metabolism. As expected, the differences between individual isolates were significant in terms of mitochondrial respiration, glycolysis and the total rate of ATP production. The differences were most pronounced with hypervirulent isolates of serotype III, ST-17, which generally activated glycolysis most strongly in stimulated macrophages.

On the other hand, isolates of ST-291 (serotype IV) and ST-498 (serotype V) induced a substantial increase in total metabolism, with high levels of glycolytic and OxPhos ATP production, suggesting that macrophages stimulated with these isolates appear to activate both major pathways of energy metabolism at approximately the same rate. In macrophages stimulated with isolates of serotype Ib, the ratio of glycolytic to OxPhos ATP production was practically unchanged compared with control due to the highest level of OxPhos ATP production observed. All of this underscores both the continued importance of OxPhos as the predominant source of ATP in GBS stimulated macrophages despite increased glycolysis and the considerable variability in metabolic response to individual serotypes. On the other hand, clinical presentation did not significantly affect any of the metabolic parameters. However, we observed a trend of higher glycolytic and OxPhos ATP production in colonizing isolates.

Interestingly, macrophages stimulated with isolates of serotype Ib had a high ratio of ECAR to OCR, although their ratio of glycolytic to OxPhos ATP production was low. This discrepancy highlights the need to quantify glycolysis rates more precisely by accounting for extracellular acidification of the medium due to CO_2_ secretion during cellular respiration by injecting rotenone and antimycin-A to block mitochondrial respiration (Mookerjee et al., 2015). This strategy also allows the quantification of oxygen consumption outside the respiratory chain. One such source of oxygen consumption in macrophages is the NADPH oxidase, which is involved in the generation of reactive oxygen species in the oxidative burst. Indeed, stimulation with most GBS isolates resulted in a significant increase in non-mitochondrial OCR, indicating a potential oxidative burst. In general, non-mitochondrial OCR correlated to some extent with cytotoxicity, as the more cytotoxic colonizing isolates (serotype IV and ST-498 of serotype V) caused the highest increase, whereas the less cytotoxic isolates (serotype III and ST-1 of serotype V) caused much smaller increase in non-mitochondrial OCR, with serotype Ia isolates being the exception. Nevertheless, the data seem to suggest that a strong, potentially excessive oxidative burst induced by some isolates may play a role in their cytotoxicity to macrophages, but further studies are needed to confirm this. Our observations confirm the important influence of metabolism on both the phenotype and macrophage effector functions, as stimulation with different isolates also resulted in different phagocytic capabilities and phenotype markers expressions. The metabolic shift toward aerobic glycolysis, characteristic of cancer cells, is also thought to be critical for the activation and exertion of pro-inflammatory functions of immune cells (Escoll and Buchrieser, 2018). Immune functions directly dependent on the switch to a Warburg-like metabolism are phagocytosis and IL-1β production, but also the acquisition of co-stimulatory capacity by dendritic cells (O'Neill and Pearce, 2016). The latter may also apply to macrophages, as our results suggest that more glycolytic macrophages also express more co-stimulatory molecules. Nevertheless, studies suggest that both glycolysis and OxPhos are critical for the exertion of immune functions and that the induction of specific metabolic programs is highly specific (both cell type and bacterial specific) (Lachmandas et al., 2016). Most importantly, our data confirm that macrophage polarization to the M1 and M2 phenotype is not so unidirectional but involves a range of macrophage activation states depending on the stimulus.

## 5. Conclusions

Overall, our data suggest that different GBS isolates affect the immune response of macrophages differently. The differences between the various isolates are evident at the level of phagocytic uptake and expression of surface markers, as well as at the level of morphology, cytotoxicity, and metabolism of infected macrophages. This suggests that the occurrence of infection is influenced not only by the immune status or susceptibility of the individual, but also by the isolate itself, as different isolates appear to have different potential to become invasive or remain colonizing. Macrophage activation and response is influenced by capsular polysaccharides and thus by bacterial serotype as well as by individual virulence factors. The results also suggest that the execution of macrophage effector functions is also influenced by the bacteria in terms of metabolic reprogramming, as macrophage functions are strongly dependent on metabolic phenotype. While colonizing isolates appear to elicit a more cytotoxic effect, invasive isolates appear to use macrophages to their advantage, making them invasive in the first place. Although differences were noted between all isolates, our data also show that hypervirulent isolates of serotype III, ST-17, which possess PI-1-2B type of pili and the protein HvgA, elicit the strongest immune response. In addition to the highest phagocytic uptake, they also induced high expression of inflammatory markers. This was also confirmed by a marked shift in energy metabolism toward glycolysis and glycolytic ATP production in macrophages stimulated with these isolates, suggesting a Warburg-like metabolism. On the other hand, serotype Ib isolates activated macrophages the least, as these macrophages had the lowest ratio of glycolytic to OxPhos ATP production, phagocytic uptake, and expression of co-stimulatory molecules.

Thus, our data confirm the existing paradigm that differences in host immune responses to GBS are due to genotype-specific differences in GBS isolates. These and similar studies could aid in the development of new prognostic strategies or improved diagnosis of GBS infections. Since targeting the host immune system could be an alternative treatment approach to combat bacteria, it is of utmost importance to better understand immune responses and their interplay with bacteria. In addition, the data obtained could prove useful for diagnostic purposes in the future, as the bacterial isolate itself could be used to infer the prognosis of the disease. Further studies both *in vitro* and *in vivo* are needed to draw more precise conclusions. Nevertheless, our results complement previous studies on the immune response to group B *Streptococci* and demonstrate for the first time the importance of the metabolic phenotype of macrophages in *Streptococcus agalactiae* infections.

## Data availability statement

The original contributions presented in the study are included in the article/Supplementary material, further inquiries can be directed to the corresponding author.

## Author contributions

LJ, AK, and AI designed the study. LJ and JR performed the experiments, data analysis, and interpretation. LJ wrote the original draft. All authors reviewed the article, contributed to the article, and approved the submitted version.

## Funding

This work was founded by the Slovenian Research Agency (ARRS) under postgraduate program and grant number P3-0083.

## Conflict of interest

The authors declare that the research was conducted in the absence of any commercial or financial relationships that could be construed as a potential conflict of interest.

## Publisher’s note

All claims expressed in this article are solely those of the authors and do not necessarily represent those of their affiliated organizations, or those of the publisher, the editors and the reviewers. Any product that may be evaluated in this article, or claim that may be made by its manufacturer, is not guaranteed or endorsed by the publisher.
