# Supplementary material for: Macrophage polarization during Streptococcus agalactiae infection is isolate specific
Source: Front Microbiol. 2023 May 4;14:1186087. doi: 10.3389/fmicb.2023.1186087 (PMC10192866; doi:10.3389/fmicb.2023.1186087)
Supplement: Supplementary file 1 [file Data_Sheet_1.docx]

**SUPPLEMENTARY MATERIAL**

**
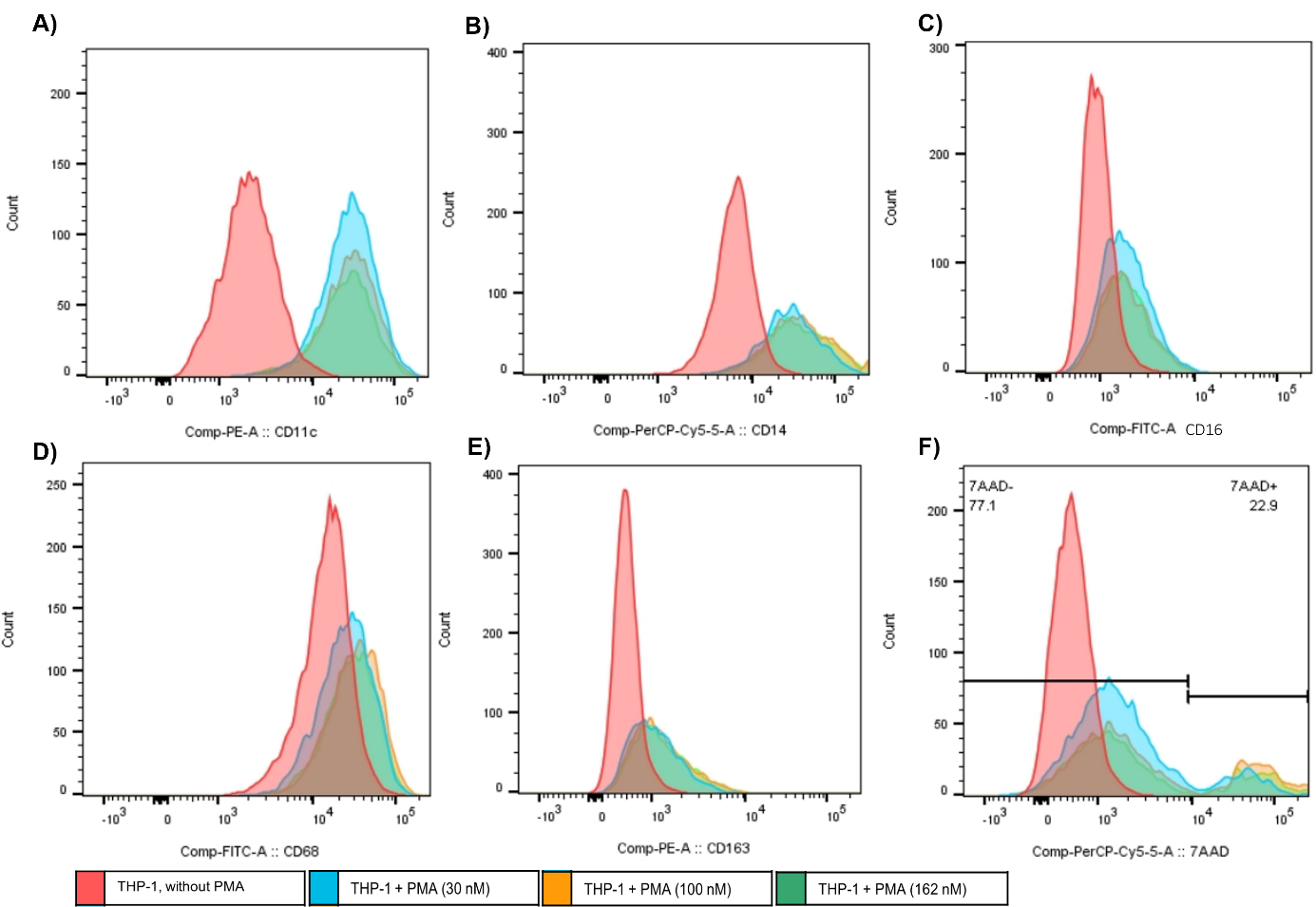
**

**Figure S1: Comparison of THP-1 differentiation with different PMA concentrations.** THP-1 monocytes were treated with 3 different concentrations of PMA (30 nM, 100 nM and 162 nM) for 24h followed by 1 day rest, and then **A)** CD11c, **B)** CD14, **C)** CD16, **D)** CD68 and **E)** CD163 expression and **F)** viability (7-AAD) were measured by flow cytometry. For further experiments, PMA with a concentration of 100 nM was chosen.
Red: non-differentiated THP-1 monocytes; blue: THP-1 macrophages, differentiated with 30 nM PMA; orange: THP-1 macrophages, differentiated with 100 nM PMA and green: THP-1 macrophages, differentiated with 162 nM PMA.


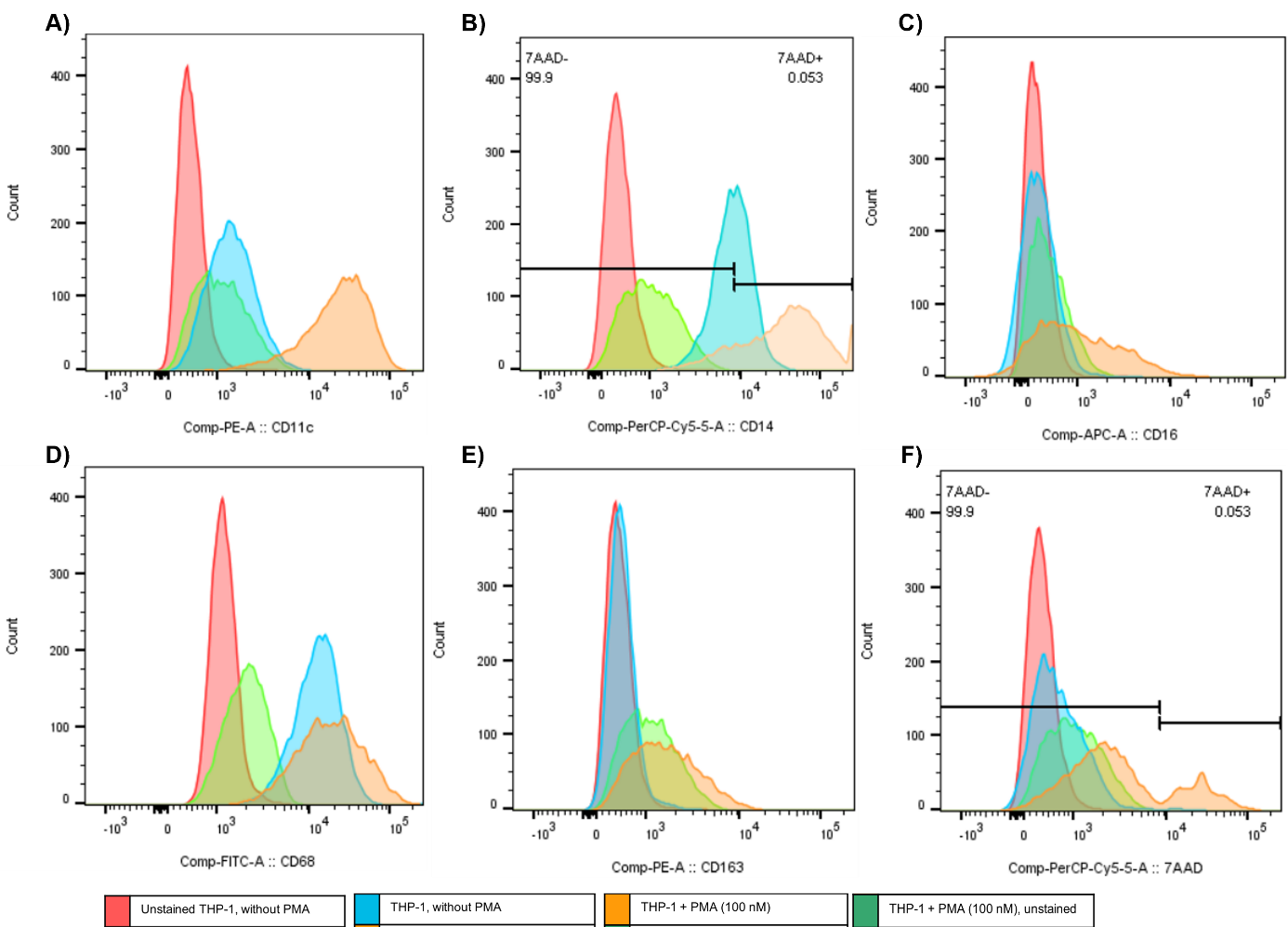


**Figure S2: THP-1 differentiation into macrophages with 100 nM PMA for 72h and 1 day rest.** THP-1 monocytes were treated with 100 nM PMA for 72H followed by 1 day rest, and then **A)** CD11c, **B)** CD14, **C)** CD16, **D)** CD68 and **E)** CD163 expression and **F)** viability (7-AAD) were measured by flow cytometry.
Red: unstained, non-differentiated THP-1 monocytes; blue: non-differentiated THP-1 monocytes; orange: THP-1 macrophages, differentiated with 100 nM PMA and green: unstained THP-1 macrophages, differentiated with 100 nM PMA.


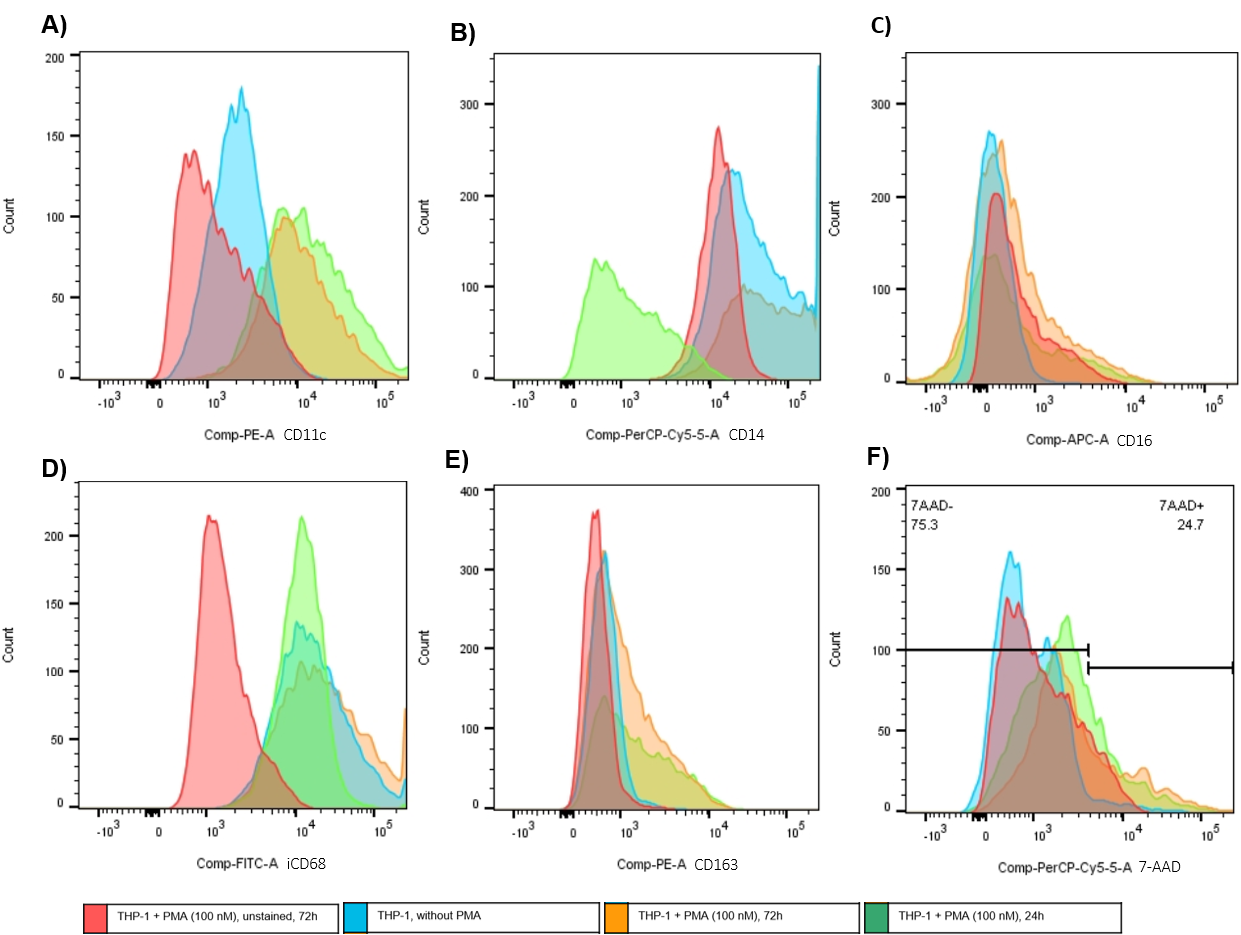


**Figure S3: THP-1 differentiation into macrophages with 100 nM PMA for 24h or 72h and 5 day rest.** THP-1 monocytes were treated with 100 nM PMA for 24h (orange) or 72h (green) followed by 5 day rest, and then **A)** CD11c, **B)** CD14, **C)** CD16, **D)** CD68 and **E)** CD163 expression and **F)** viability (7-AAD) were measured by flow cytometry.
Red: unstained THP-1 macrophages, differentiated with 100 nM PMA for 72h; blue: non-differentiated THP-1 macrophages; orange: THP-1 macrophages, differentiated with 100 nM PMA for 72h and green: THP-1 macrophages, differentiated with 100 nM PMA for 24h.

**
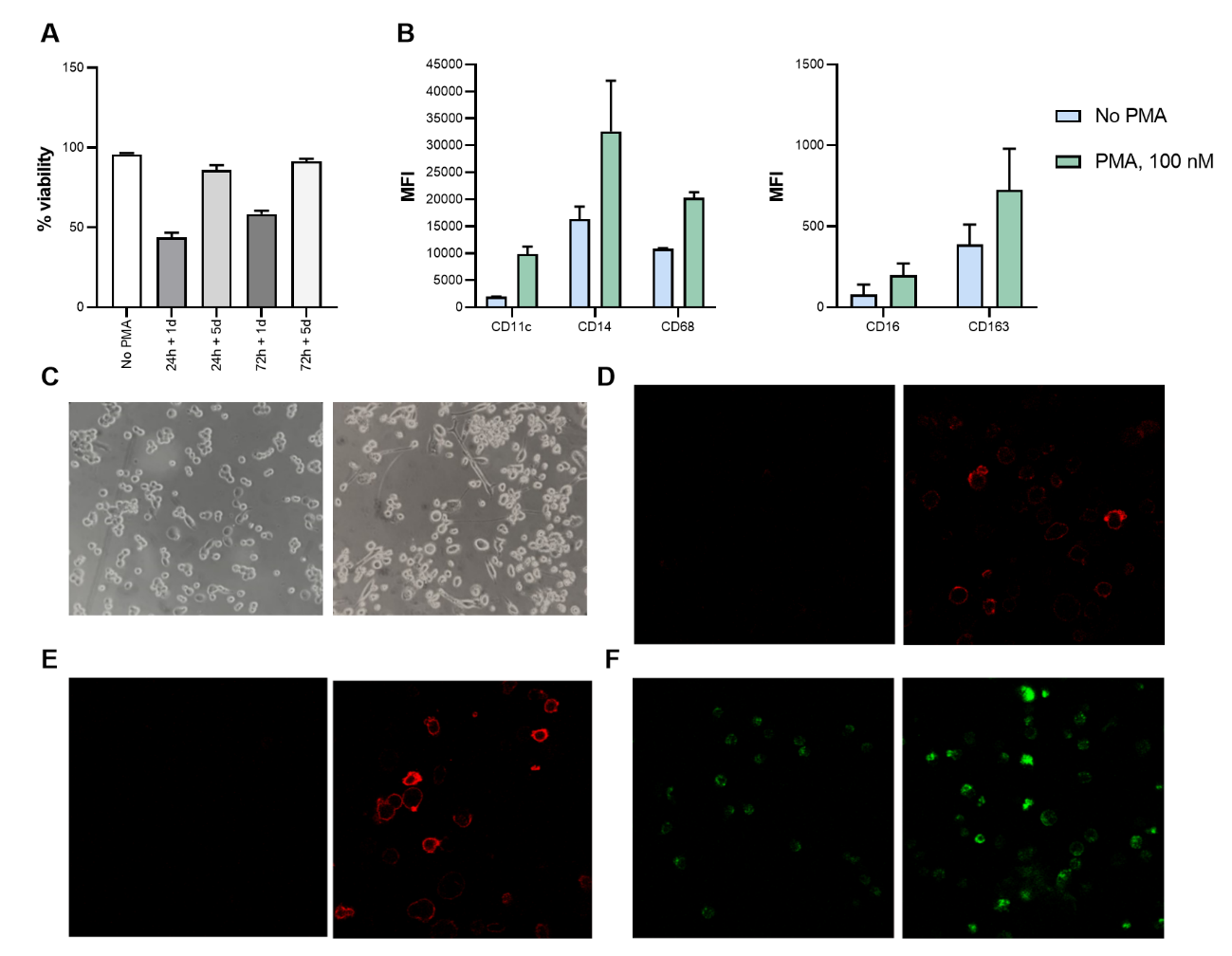
**

**Figure S4: Optimization of THP-1 differentiation into macrophages. A)** THP-1 monocytes were differentiated into macrophages with 100 nM PMA for different periods of time, and then viability was measured by 7-AAD staining. **B)** CD11c, CD14, CD68, CD16, and CD163 expression on THP-1 macrophages differentiated with 100 nm PMA for 72h and 5 day rest compared to non-differentiated THP-1 monocytes. Data from three independent biological replicates are shown as mean ± SEM. **C)** Morphology of THP-1 macrophages, differentiated with 100 nM PMA for 24h and 1 day rest (left) or 72h and 5 day rest (right) by light microscopy (200x magnification). **D-F)** Expression of **D)** CD11c (red, right), **E)** CD14 (red, right) and **F)** CD68 (green, right) on THP-1 macrophages, differentiated with 100 nM PMA for 72h and 5 day rest compared to non-differentiated monocytes (left), by confocal microscopy (600x magnification, scale bar: 10 µm).


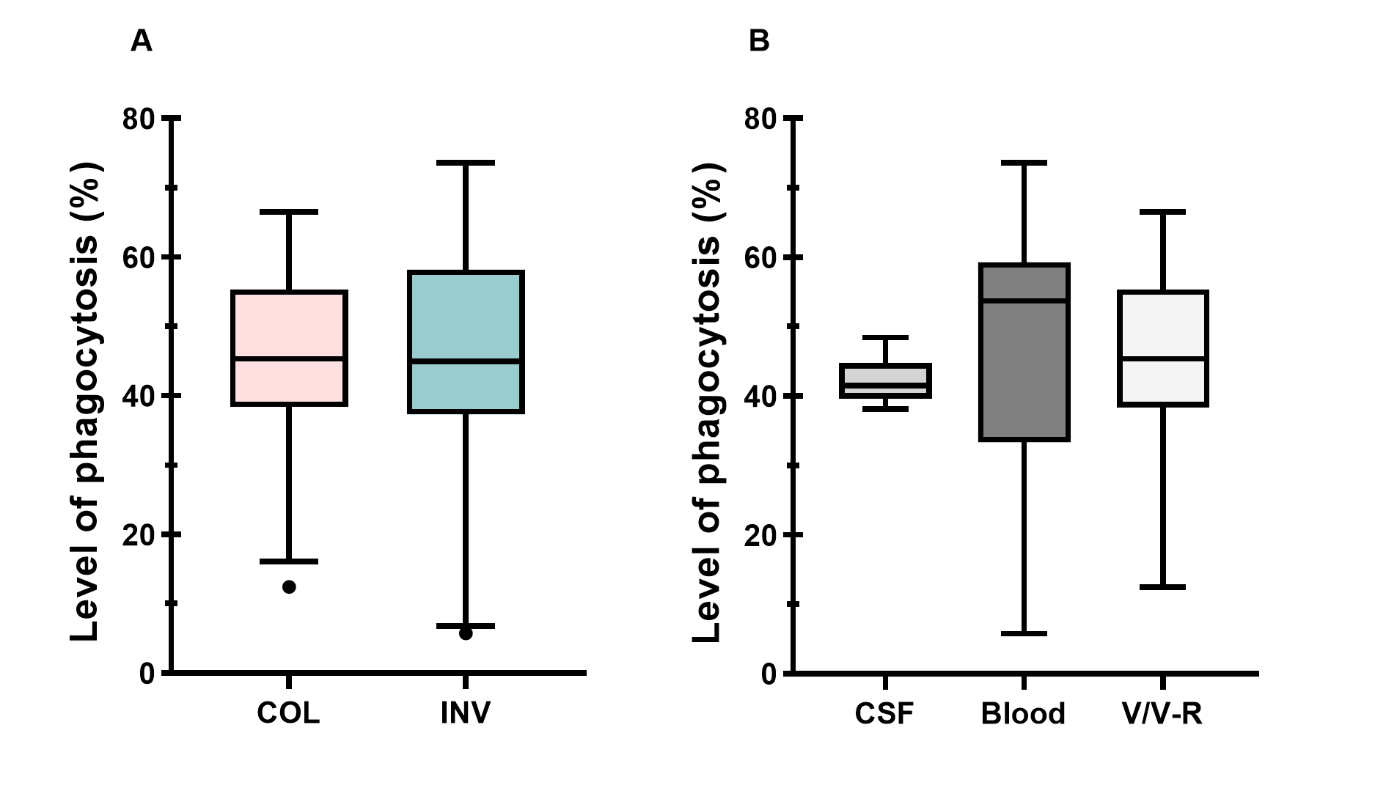


**Figure S5: Phagocytosis of 12 different GBS isolates**. Boxplots show phagocytic uptake of GBS isolates after a 3-hour infection period according to the **A)** type of clinical presentation (COL – colonizing and INV – invasive) and **B)** type of specimen (CSF – cerebrospinal fluid, V/V-R – vagina/vagina-rectum). Data from three independent biological replicates (n = 3) are shown. The bottom and top borders represent the 25^th^ and 75^th^ percentile, respectively, the middle line represents the median and the whiskers indicate variability outside the middle quartiles. Statistical differences were determined by un-paired t-test or one-way ANOVA with Tukey’s *post-hoc* test.


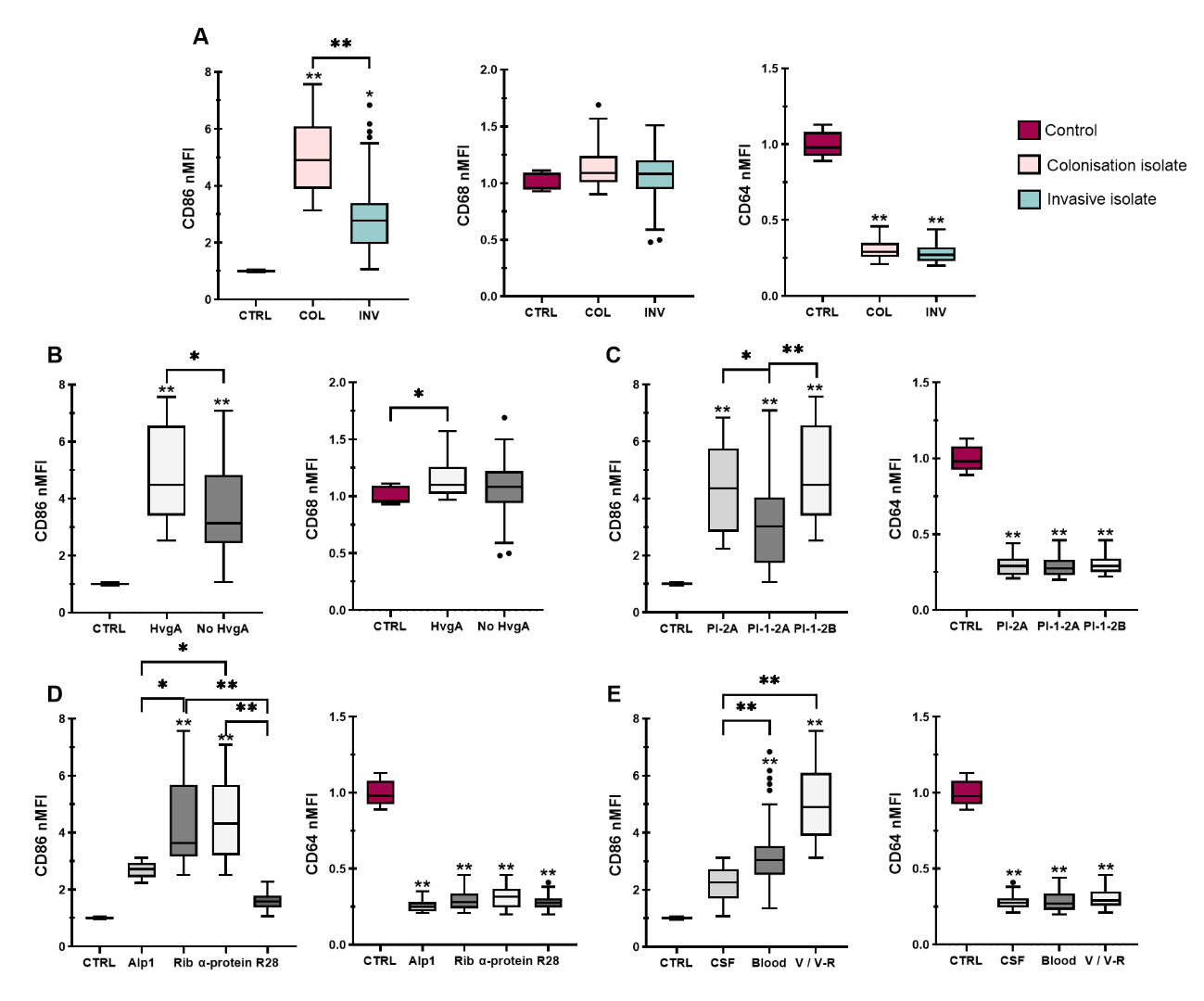


**Figure S6: Expression of macrophage phenotype markers 3 days after GBS infection**. Expression of CD64, CD86 and CD68 on THP-1 macrophages after stratification of data by **A)** clinical presentation (CTRL – negative control, COL – colonizing, INV – invasive), **B)** presence or absence of virulence factor protein HvgA, **C)** pilus type, **D)** Alp proteins and **E)** specimen (CSF – cerebrospinal fluid, V/V-R – vagina/vagina-rectum). Data from three independent biological replicates, each with three technical replicates, are shown (n = 9). *P ≤ 0.05 and **P ≤ 0.01 versus non-stimulated control macrophages (unless otherwise noted), as determined by the Kruskal-Wallis test with Dunn’s *post-hoc* test.


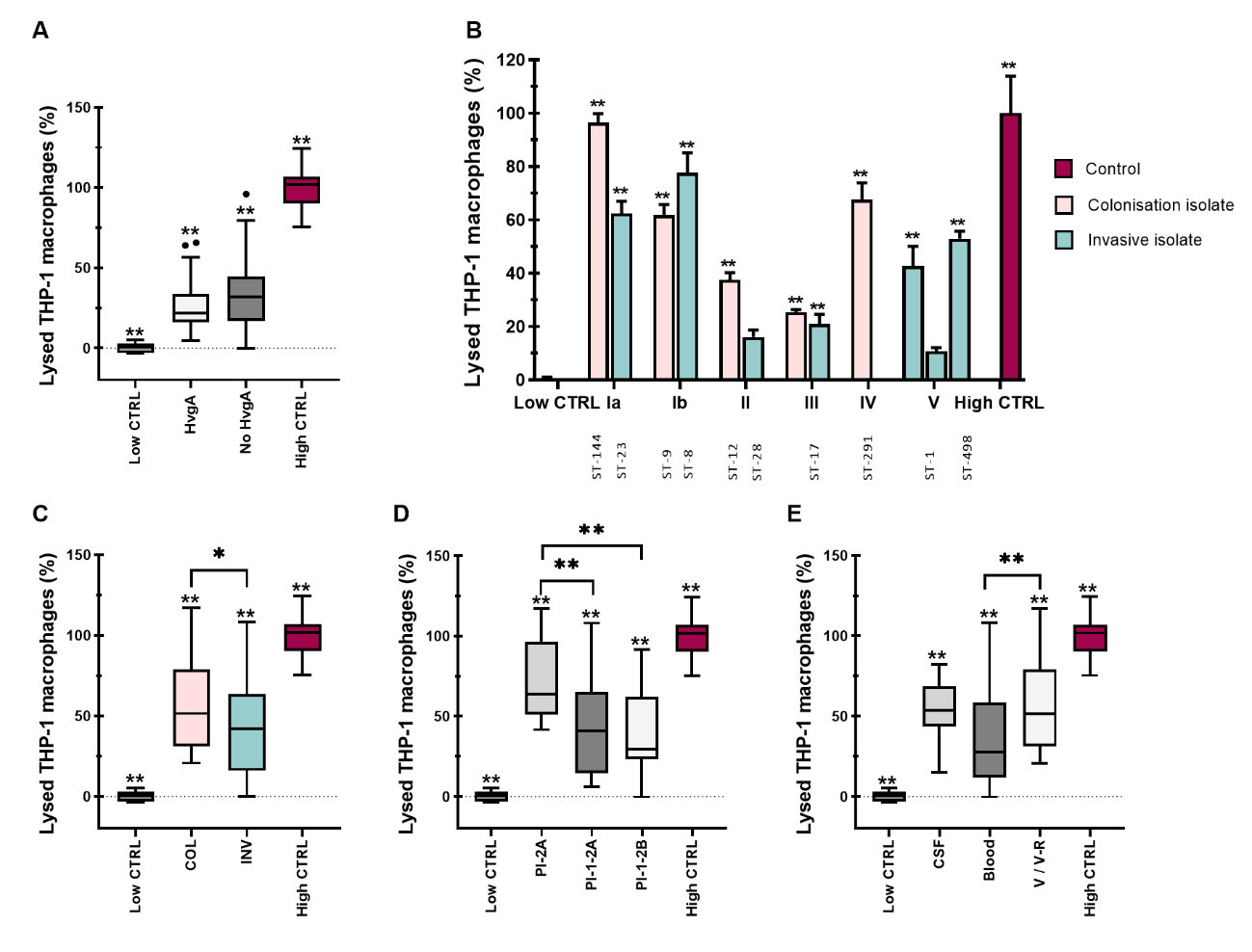


**Figure S7: GBS mediated cell cytotoxicity**. Macrophage lysis after stimulation with 12 different GBS isolates. **A)** Comparison of GBS cytotoxicity (MOI 10:1) according to HvgA protein.
**B-E)** GBS cytotoxicity at a MOI of 20 was determined by LDH release. **B)** Comparison of cytotoxicity of GBS isolates, belonging to 6 different serotypes and 10 different ST. Data from three independent biological replicates, each with three technical replicates, are shown as mean ± SEM (n = 9). All isolates (except for isolate of ST-144) induced significantly lower cytotoxicity compared with the high control. *P ≤ 0.05 and **P ≤ 0.01 versus low control as determined by one-way ANOVA and *post-hoc* Šidák’s test. Statistical differences between individual isolates are shown in Table S3B.
GBS mediated cell cytotoxicity according to **C)** clinical presentation (COL – colonizing, INV – invasive), **D)** pilus type and **E)** specimen (CSF – cerebrospinal fluid, V/V-R – vagina/vagina-rectum). *P ≤ 0.05 and **P ≤ 0.01 by un-paired t-test or Kruskal-Wallis test, followed by *post-hoc* Dunn’s multiple-comparison test, respectively.


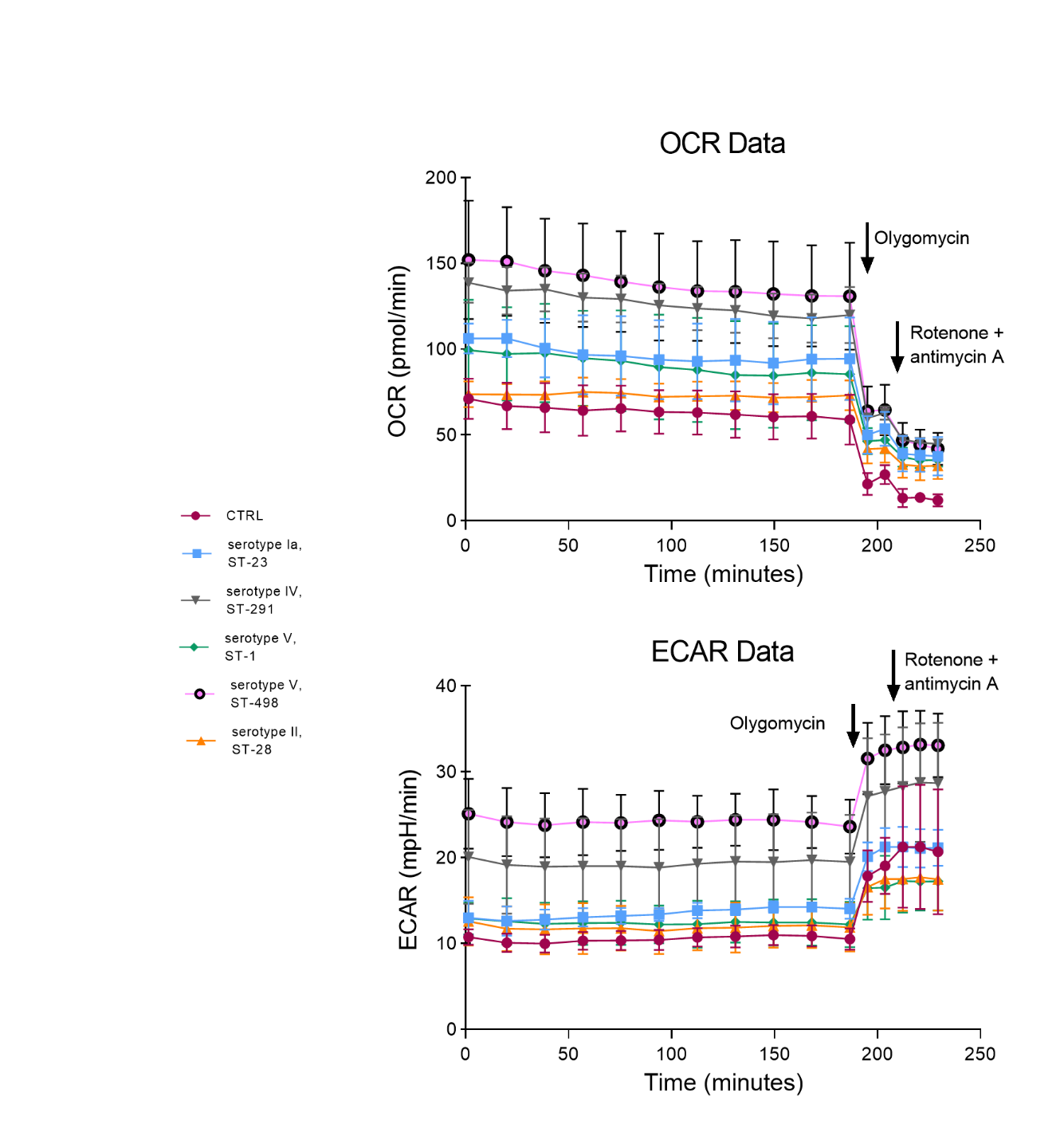


**Figure S8: Injection strategy for oligomycin and rotenone plus antimycin-A inhibitors.** Graphs of a representative experiment show the time course of OCR (above) and ECAR (bellow) measurements and injection strategy. The timing and sequence is standard for the Seahorse Real-Time ATP Rate Assay (Agilent, USA).


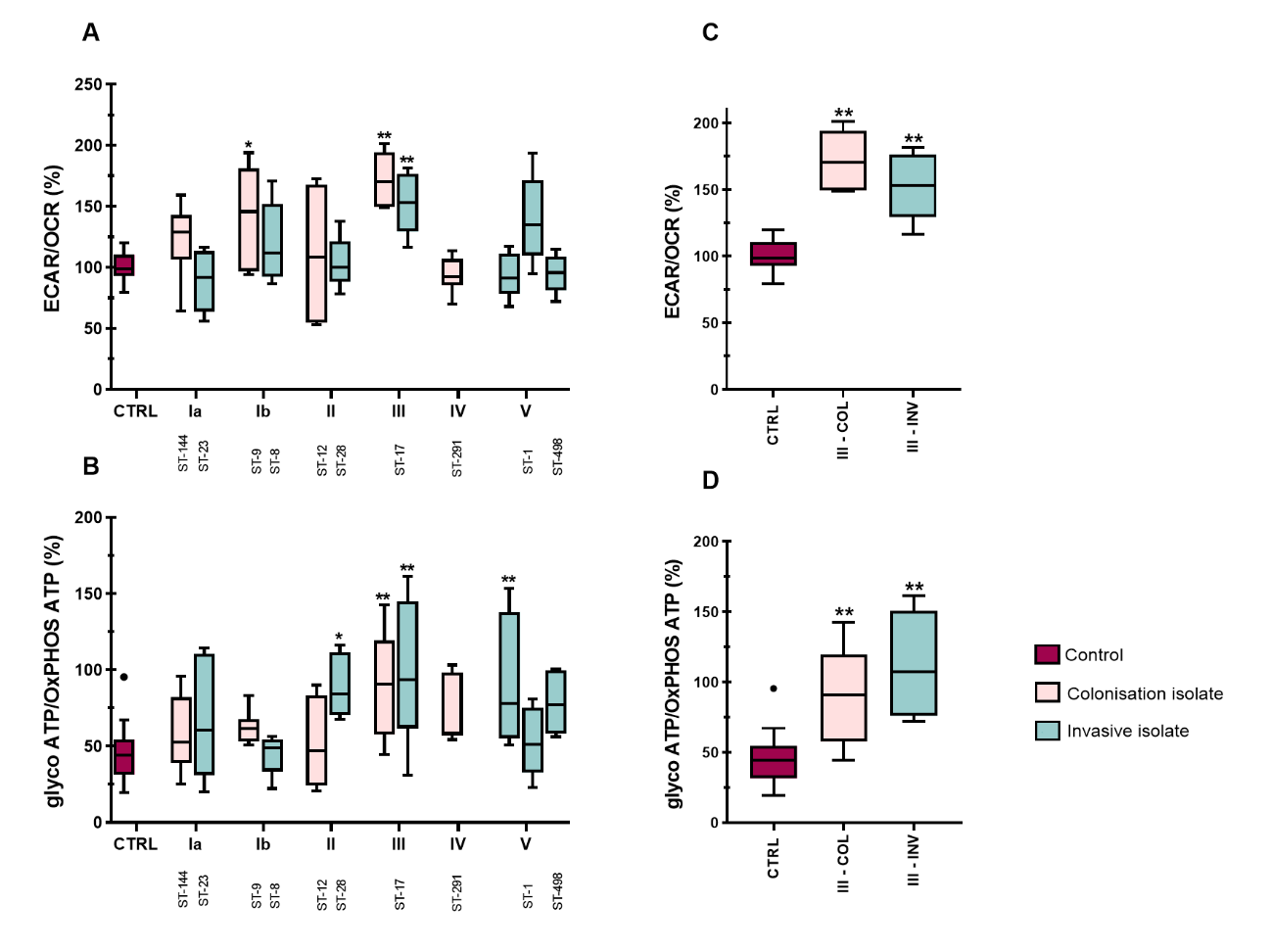


**Figure S9: Macrophages stimulated with different GBS isolates differ in the ratios of glycolysis to mitochondrial respiration.** Basal ECAR and OCR were measured 4 hours after infection with 12 various GBS isolates, and ATP production from OxPhos and glycolysis was determined according to Seahorse Real Time ATP Production Rate Assay. Data from two independent experiments, each with 3 technical replicates, were obtained (n = 6) and normalized to non-stimulated control macrophages. **A**-**B**) The ratio of **A)** ECAR to OCR and **B)** glycolytic to OxPhos ATP production for macrophages stimulated with individual isolates. **C-D)** The ratio of **C**) ECAR to OCR and **D)** glycolytic to OxPhos ATP production for macrophages stimulated with colonizing or invasive isolates of serotype III, ST-17, compared with non-stimulated control macrophages. *P ≤ 0.05 and **P ≤ 0.01 versus non-stimulated control macrophages as determined by one-way ANOVA with Šidák’s (**A**-**B**) or Tukey’s (**C**-**D**) *post-hoc* test. Statistical differences for glycolysis ATP / OxPHOS ATP and ECAR / OCR between individual isolates are shown in Tables S6 and S7.


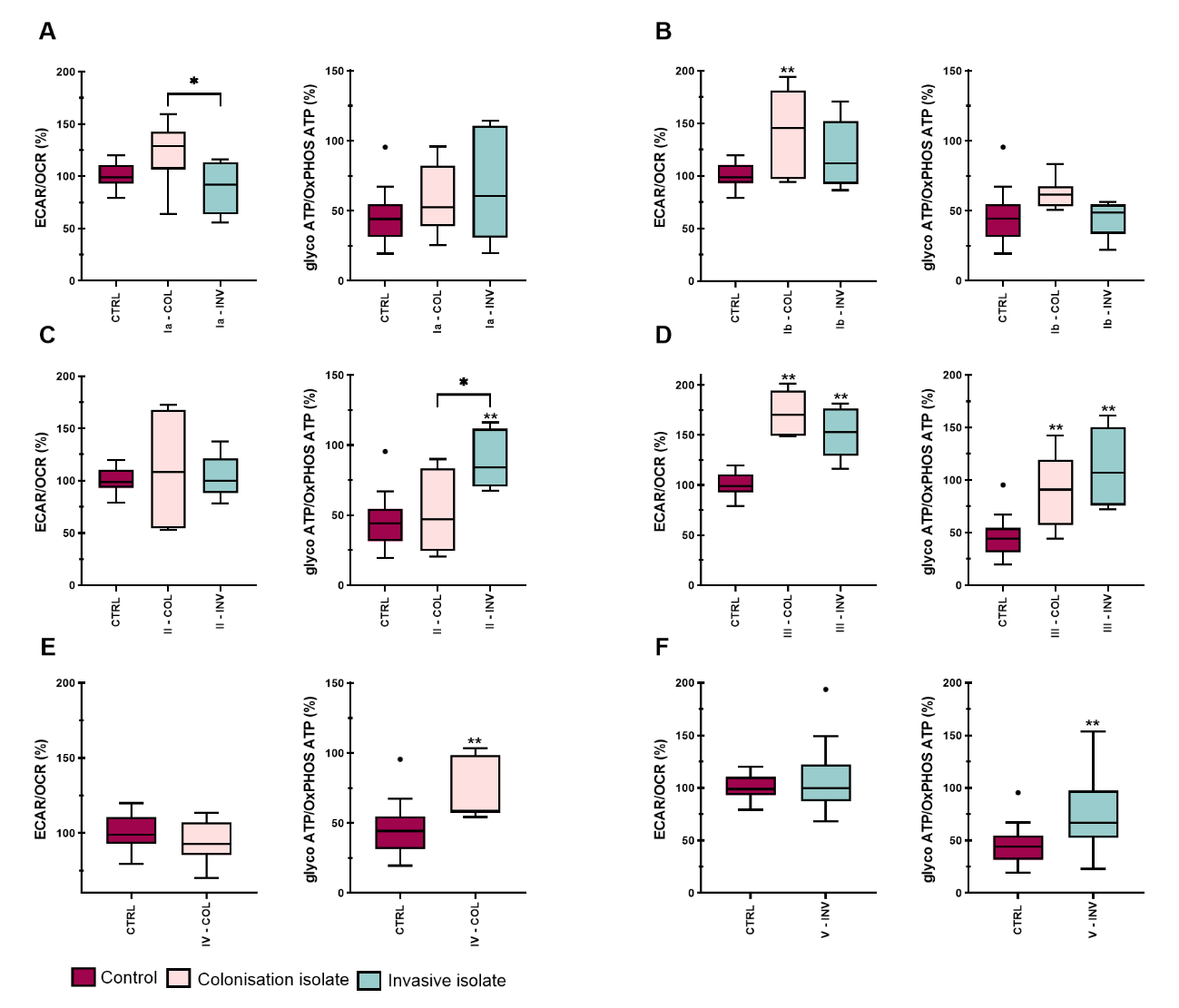


**Figure S10: Ratio of ECAR to OCR and glycolysis ATP to OxPhos ATP in macrophages, stimulated with individual isolates, compared with non-stimulated control THP-1 macrophages**. ECAR and OCR were measured by Seahorse Extracellular Flux Analyzer 4 hours after infection and ATP production was determined using Seahorse Real-Time ATP Rate Assay. Boxplots represent ratios of glycolysis ATP to OxPhos ATP and ECAR to OCR for macrophages, stimulated with **A)** colonizing and invasive isolates of serotype Ia, **B)** colonizing and invasive isolates of serotype Ib, **C)** colonizing and invasive isolates of serotype II, **D)** colonizing and invasive isolates of serotype III, **E)** colonizing isolate of serotype IV and **F)** invasive isolates of serotype V. CTRL – control, non-stimulated macrophages, COL – colonizing, INV – invasive. Asterisks above the boxplots represent statistically significant differences versus control macrophages and were obtained by ANOVA and *post-hoc* Tukey’s test, where *P ≤ 0.05 and **P ≤ 0.01 (n = 6).

**
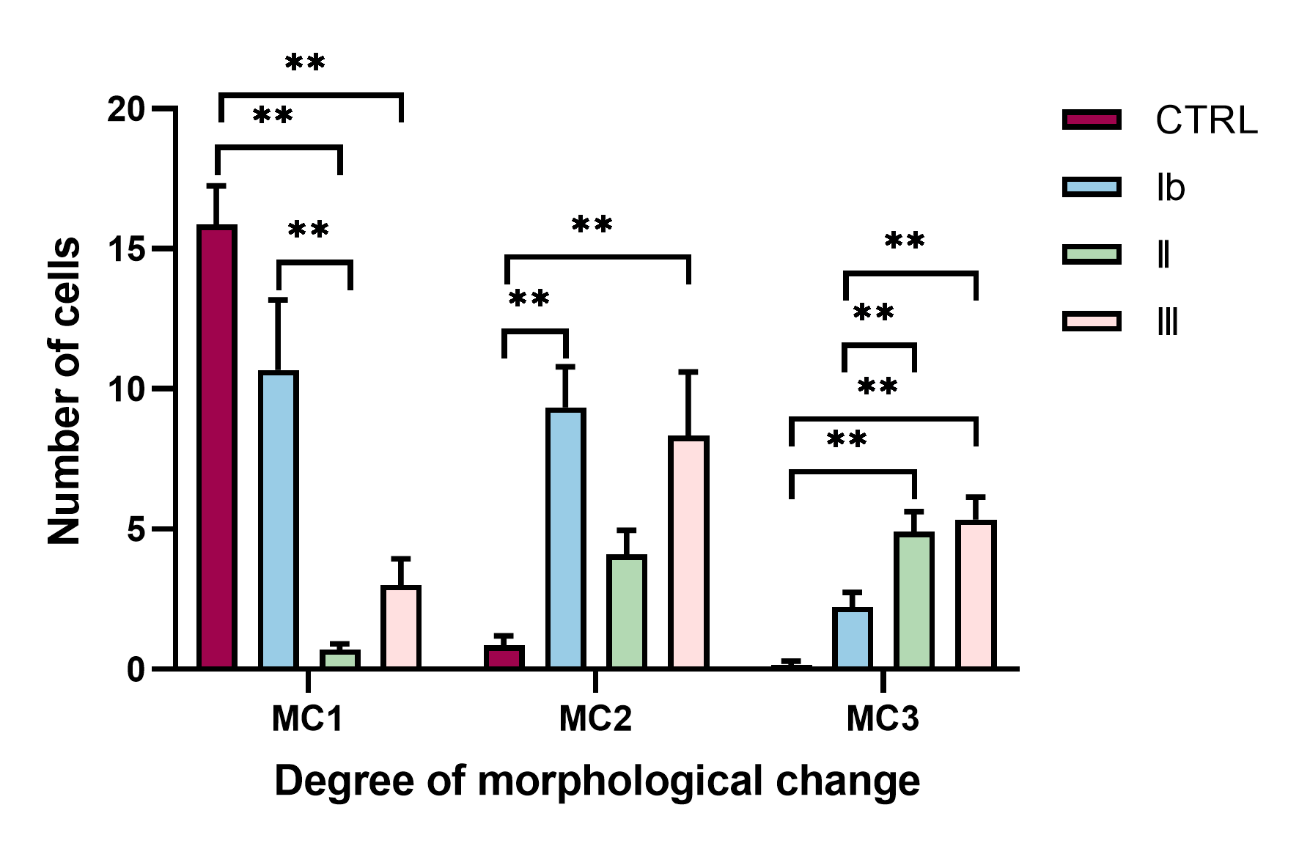
**

**Figure S11: Morphological changes of THP-1 macrophages after infection with three different GBS isolates.** Macrophages were stimulated with GBS isolates for 3 days and observed for morphological changes with a confocal microscope. MC1 indicates round, unbranched, non-activated macrophages. MC2 indicates moderately activated, elongated macrophages but without obvious pseudopodia. MC3 indicates highly differentiated macrophages with numerous pseudopodia. Morphological changes were quantified in a blinded fashion and number of cells with altered morphology was enumerated. Representative data of three technical replicates are shown as mean ± SEM. Statistical differences (*P ≤ 0.05 and **P ≤ 0.01) were determined by one-way ANOVA or Kruskal-Wallis test.

**Video 1: Real-time microscopy of control, non-stimulated THP-1 macrophages.** During a 3-hour incubation period, 360 images were acquired, i.e. 1 image per 30 seconds.

**Video 2: Real-time microscopy of macrophages stimulated with colonizing isolate of serotype Ia.** During a 3-hour incubation period, 360 images were acquired, i.e. 1 image per 30 seconds.

**Video 3: Real-time microscopy of macrophages stimulated with invasive isolate of serotype Ib.** During a 3-hour incubation period, 360 images were acquired, i.e. 1 image per 30 seconds.

**Video 4: Real-time microscopy of macrophages stimulated with invasive isolate of serotype III.** During a 3-hour incubation period, 360 images were acquired, i.e. 1 image per 30 seconds.

**Table S1: Statistical differences between individual isolates for phagocytosis, as determined by ANOVA with Šidák’s *post-hoc* test.**

| **GBS isolates** | **Šidák's multiple comparisons test (adjusted p-value)** |  | **GBS isolates** | **Šidák's multiple comparisons test (adjusted p-value)** |
| --- | --- | --- | --- | --- |
| 203 (ST-8) vs. 231 (ST-17) | <0.0001 |  | 10276 (ST-9) vs. 8422 (ST-291) | 0.0004 |
| 203 (ST-8) vs. 9731 (ST-17) | <0.0001 |  | 10276 (ST-9) vs. 6 (ST-1) | 0.003 |
| 203 (ST-8) vs. 211 (ST-28) | <0.0001 |  | 10276 (ST-9) vs. 123 (ST-498) | <0.0001 |
| 203 (ST-8) vs. 7339 (ST-12) | <0.0001 |  | 231 (ST-17) vs. 7339 (ST-12) | 0.0043 |
| 203 (ST-8) vs. 229 (ST-23) | 0.0003 |  | 231 (ST-17) vs. 229 (ST-23) | 0.0006 |
| 203 (ST-8) vs. 9427 (ST-144) | <0.0001 |  | 231 (ST-17) vs. 9427 (ST-144) | 0.0087 |
| 203 (ST-8) vs. 8422 (ST-291) | <0.0001 |  | 231 (ST-17) vs. 8422 (ST-291) | 0.0245 |
| 203 (ST-8) vs. 6 (ST-1) | <0.0001 |  | 231 (ST-17) vs. 6 (ST-1) | 0.003 |
| 203 (ST-8) vs. 104 (ST-1) | 0.0018 |  | 231 (ST-17) vs. 104 (ST-1) | <0.0001 |
| 203 (ST-8) vs. 123 (ST-498) | <0.0001 |  | 9731 (ST-17) vs. 7339 (ST-12) | 0.0482 |
| 10276 (ST-9) vs. 231 (ST-17) | <0.0001 |  | 9731 (ST-17) vs. 229 (ST-23) | 0.0073 |
| 10276 (ST-9) vs. 9731 (ST-17) | <0.0001 |  | 9731 (ST-17) vs. 6 (ST-1) | 0.0347 |
| 10276 (ST-9) vs. 211 (ST-28) | <0.0001 |  | 9731 (ST-17) vs. 104 (ST-1) | 0.0012 |
| 10276 (ST-9) vs. 7339 (ST-12) | 0.0021 |  | 229 (ST-23) vs. 123 (ST-498) | 0.0415 |
| 10276 (ST-9) vs. 229 (ST-23) | 0.0149 |  | 104 (ST-1) vs. 123 (ST-498) | 0.007 |
| 10276 (ST-9) vs.9427 (ST-144) | 0.001 |  |  |  |

**Table S2: Statistical differences between individual isolates in expression of the macrophage markers CD64 (A), CD80 (B), CD86 (C), CD68 (D), CD163 (E) and CD206 (F), as determined by one-way ANOVA with Šidák’s *post-hoc* test.**

**Table S2A: Expression of CD64**

| **GBS isolates** | **Šidák's multiple comparisons test (adjusted p-value)** |
| --- | --- |
| 211 (ST-28) vs. 9731 (ST-17) | 0.0152 |
| 211 (ST-28) vs. 123 (ST-498) | 0.0453 |
| 104 (ST-1) vs. 123 (ST-498) | 0.0221 |

**Table S2B: Expression of CD80**

| **GBS isolates** | **Šidák's multiple comparisons test (adjusted p-value)** |  | **GBS isolates** | **Šidák's multiple comparisons test (adjusted p-value)** |
| --- | --- | --- | --- | --- |
| 231 (ST-17) vs. 203 (ST-8) | <0.0001 |  | 7339 (ST-12) vs. 9731 (ST-17) | 0.0004 |
| 9731 (ST-17) vs. 203 (ST-8) | 0.0003 |  | 6 (ST-1) vs. 9731 (ST-17) | <0.0001 |
| 211 (ST-28) vs. 203 (ST-8) | <0.0001 |  | 7339 (ST-12) vs. 211 (ST-28) | 0.0042 |
| 7339 (ST-12) vs. 203 (ST-8) | <0.0001 |  | 229 (ST-23) vs. 211 (ST-28) | 0.005 |
| 9427 (ST-144) vs. 203 (ST-8) | 0.0034 |  | 6 (ST-1) vs. 211 (ST-28) | <0.0001 |
| 8422 (ST-291) vs. 203 (ST-8) | <0.0001 |  | 229 (ST-23) vs. 7339 (ST-12) | <0.0001 |
| 104 (ST-1) vs. 203 (ST-8) | 0.0063 |  | 9427 (ST-144) vs. 7339 (ST-12) | <0.0001 |
| 123 (ST-498) vs. 203 (ST-8) | 0.0016 |  | 8422 (ST-291) vs. 7339 (ST-12) | 0.0484 |
| 231 (ST-17) vs. 10276 (ST-9) | 0.0043 |  | 6 (ST-1) vs. 7339 (ST-12) | <0.0001 |
| 9731 (ST-17) vs. 10276 (ST-9) | 0.0121 |  | 104 (ST-1) vs. 7339 (ST-12) | <0.0001 |
| 211 (ST-28) vs. 10276 (ST-9) | 0.001 |  | 123 (ST-498) vs. 7339 (ST-12) | <0.0001 |
| 7339 (ST-12) vs. 10276 (ST-9) | <0.0001 |  | 8422 (ST-291) vs. 229 (ST-23) | 0.0002 |
| 8422 (ST-291) vs. 10276 (ST-9) | <0.0001 |  | 6 (ST-1) vs. 9427 (ST-144) | 0.0003 |
| 123 (ST-498) vs. 10276 (ST-9) | 0.0475 |  | 6 (ST-1) vs. 8422 (ST-291) | <0.0001 |
| 7339 (ST-12) vs. 231 (ST-17) | 0.0013 |  | 104 (ST-1) vs. 6 (ST-1) | 0.0003 |
| 229 (ST-23) vs. 231 (ST-17) | 0.0233 |  | 123 (ST-498) vs. 6 (ST-1) | <0.0001 |
| 6 (ST-1) vs. 231 (ST-17) | <0.0001 |  |  |  |

**Table S2C: Expression of CD86**

| **GBS isolates** | **Šidák's multiple comparisons test (adjusted p-value)** |  | **GBS isolates** | **Šidák's multiple comparisons test (adjusted p-value)** |
| --- | --- | --- | --- | --- |
| 10276 vs. 203 (ST-8) | 0.0477 |  | 8422 (ST-291) vs. 9731 (ST-17) | <0.0001 |
| 9731 (ST-17) vs. 203 (ST-8) | <0.0001 |  | 6 (ST-1) vs. 9731 (ST-17) | <0.0001 |
| 7339 (ST-12) vs. 203 (ST-8) | <0.0001 |  | 104 (ST-1) vs. 9731 (ST-17) | <0.0001 |
| 9427 (ST-144) vs. 203 (ST-8) | 0.0007 |  | 7339 (ST-12) vs. 211 (ST-28) | <0.0001 |
| 8422 (ST-291) vs. 203 (ST-8) | 0.0002 |  | 9427 (ST-144) vs. 211 (ST-28) | 0.0095 |
| 104 (ST-1) vs. 203 (ST-8) | 0.0187 |  | 8422 (ST-291) vs. 211 (ST-28) | 0.0028 |
| 123 (ST-498) vs. 203 (ST-8) | <0.0001 |  | 6 (ST-1) vs. 211 (ST-28) | 0.0046 |
| 9731 (ST-17) vs. 10276 (ST-9) | <0.0001 |  | 104 (ST-1) vs. 211 (ST-28) | 0.0006 |
| 7339 (ST-12) vs. 10276 (ST-9) | 0.0067 |  | 123 (ST-498) vs. 211 (ST-28) | <0.0001 |
| 229 (ST-23) vs. 10276 (ST-9) | 0.0218 |  | 229 (ST-23) vs. 7339 (ST-12) | <0.0001 |
| 6 (ST-1) vs. 10276 (ST-9) | <0.0001 |  | 6 (ST-1) vs. 7339 (ST-12) | <0.0001 |
| 104 (ST-1) vs. 10276 (ST-9) | <0.0001 |  | 104 (ST-1) vs. 7339 (ST-12) | <0.0001 |
| 123 (ST-498) vs. 10276 (ST-9) | 0.0001 |  | 9427 (ST-144) vs. 229 (ST-23) | 0.0001 |
| 9731 (ST-17) vs. 231 (ST-17) | <0.0001 |  | 8422 (ST-291) vs. 229 (ST-23) | <0.0001 |
| 7339 (ST-12) vs. 231 (ST-17) | <0.0001 |  | 104 (ST-1) vs. 229 (ST-23) | 0.0173 |
| 9427 (ST-144) vs. 231 (ST-17) | 0.0167 |  | 123 (ST-498) vs. 229 (ST-23) | <0.0001 |
| 8422 (ST-291) vs. 231 (ST-17) | 0.005 |  | 6 (ST-1) vs. 9427 (ST-144) | <0.0001 |
| 6 (ST-1) vs. 231 (ST-17) | 0.0042 |  | 104 (ST-1) vs. 9427 (ST-144) | <0.0001 |
| 104 (ST-1) vs. 231 (ST-17) | 0.0005 |  | 123 (ST-498) vs. 9427 (ST-144) | 0.0039 |
| 123 (ST-498) vs. 231 (ST-17) | <0.0001 |  | 6 (ST-1) vs. 8422 (ST-291) | <0.0001 |
| 211 (ST-28) vs. 9731 (ST-17) | <0.0001 |  | 104 (ST-1) vs. 8422 (ST-291) | <0.0001 |
| 7339 (ST-12) vs. 9731 (ST-17) | 0.0182 |  | 123 (ST-498) vs. 8422 (ST-291) | 0.0094 |
| 229 (ST-23) vs. 9731 (ST-17) | <0.0001 |  | 123 (ST-498) vs. 6 (ST-1) | <0.0001 |
| 9427 (ST-144) vs. 9731 (ST-17) | <0.0001 |  | 123 (ST-498) vs. 104 (ST-1) | <0.0001 |

**Table S2D: Expression of CD68**

| **GBS isolates** | **Šidák's multiple comparisons test (adjusted p-value)** |
| --- | --- |
| 231 (ST-17) vs. 203 (ST-8) | 0.0467 |
| 211 (ST-28) vs. 203 (ST-8) | 0.0198 |
| 6 (ST-1) vs. 203 (ST-8) | <0.0001 |
| 211 (ST-28) vs. 10276 (ST-9) | 0.0445 |
| 6 (ST-1) vs. 231 (ST-17) | <0.0001 |
| 6 (ST-1) vs. 9731 (ST-17) | <0.0001 |
| 6 (ST-1) vs. 211 (ST-28) | <0.0001 |
| 6 (ST-1) vs. 7339 (ST-12) | <0.0001 |
| 6 (ST-1) vs. 229 (ST-23) | <0.0001 |
| 6 (ST-1) vs. 9427 (ST-144) | <0.0001 |
| 6 (ST-1) vs. 8422 (ST-291) | <0.0001 |
| 104 (ST-1) vs. 6 (ST-1) | <0.0001 |
| 123 (ST-498) vs. 6 (ST-1) | <0.0001 |

**Table S2E: Expression of CD163**

| **GBS isolates** | **Šidák's multiple comparisons test (adjusted p-value)** |
| --- | --- |
| 7339 (ST-12) vs. 203 (ST-8) | 0.0041 |
| 6 (ST-1) vs. 203 (ST-8) | 0.0053 |
| 7339 (ST-12) vs. 10276 (ST-9) | 0.0055 |
| 6 (ST-1) vs. 10276 (ST-9) | 0.0071 |
| 104 (ST-1) vs. 9731 (ST-17) | 0.0293 |
| 7339 (ST-12) vs. 211 (ST-28) | 0.0276 |
| 6 (ST-1) vs. 211 (ST-28) | 0.0343 |
| 104 (ST-1) vs. 7339 (ST-12) | <0.0001 |
| 104 (ST-1) vs. 9427 (ST-144) | 0.0044 |
| 104 (ST-1) vs. 8422 (ST-291) | 0.0021 |
| 104 (ST-1) vs. 6 (ST-1) | <0.0001 |
| 123 (ST-498) vs. 6 (ST-1) | 0.0369 |
| 123 (ST-498) vs. 104 (ST-1) | 0.0352 |

**Table S2F: Expression of CD206**

| **GBS isolates** | **Šidák's multiple comparisons test (adjusted p-value)** |
| --- | --- |
| 7339 (ST-12) vs. 203 (ST-8) | 0.0275 |
| 6 (ST-1) vs. 203 (ST-8) | 0.0001 |
| 6 (ST-1) vs. 10276 (ST-9) | 0.0005 |
| 6 (ST-1) vs. 231 (ST-17) | 0.0011 |
| 6 (ST-1) vs. 211 (ST-28) | 0.0011 |
| 104 (ST-1) vs. 7339 (ST-12) | 0.0032 |
| 6 (ST-1) vs. 229 (ST-23) | 0.0071 |
| 6 (ST-1) vs. 9427 (ST-144) | 0.0496 |
| 104 (ST-1) vs. 9427 (ST-144) | 0.0467 |
| 6 (ST-1) vs. 8422 (ST-291) | 0.0361 |
| 104 (ST-1) vs. 8422 (ST-291) | 0.0365 |
| 104 (ST-1) vs. 6 (ST-1) | <0.0001 |
| 123 (ST-498) vs. 6 (ST-1) | 0.0034 |

**Table S3: Statistical differences between individual isolates according to LDH release for MOI of 10 (A) and MOI of 20 (B), as determined by one-way ANOVA with Šidák’s *post-hoc* test.**

**Table S3A: LDH for MOI of 10**

| **GBS isolates** | **Šidák's multiple comparisons test (adjusted p-value)** |  | **GBS isolates** | **Šidák's multiple comparisons test (adjusted p-value)** |
| --- | --- | --- | --- | --- |
| 231 (ST-17) vs. 203 (ST-8) | <0.0001 |  | 7339 (ST-12) vs. 211 (ST-28) | <0.0001 |
| 9731 (ST-17) vs. 203 (ST-8) | <0.0001 |  | 229 (ST-23) vs. 211 (ST-28) | <0.0001 |
| 211 (ST-28) vs. 203 (ST-8) | <0.0001 |  | 9427 (ST-144) vs. 211 (ST-28) | <0.0001 |
| 9427 (ST-144) vs. 203 (ST-8) | <0.0001 |  | 8422 (ST-291) vs. 211 (ST-28) | <0.0001 |
| 6 (ST-1) vs. 203 (ST-8) | 0.0001 |  | 9427 (ST-144) vs. 7339 (ST-12) | <0.0001 |
| 104 (ST-1) vs. 203 (ST-8) | <0.0001 |  | 8422 (ST-291) vs. 7339 (ST-12) | 0.0018 |
| 231 (ST-17) vs. 10276 (ST-9) | <0.0001 |  | 104 (ST-1) vs. 7339(ST-12) | <0.0001 |
| 9731 (ST-17) vs. 10276 (ST-9) | <0.0001 |  | 9427 (ST-144) vs. 229 (ST-23) | <0.0001 |
| 211 (ST-28) vs. 10276 (ST-9) | <0.0001 |  | 6 (ST-1) vs. 229 (ST-23) | 0.0016 |
| 9427 (ST-144) vs. 10276 (ST-9) | <0.0001 |  | 104 (ST-1) vs. 229 (ST-23) | <0.0001 |
| 6 (ST-1) vs. 10276 (ST-9) | 0.0003 |  | 8422 (ST-291) vs. 9427 (ST-144) | <0.0001 |
| 104 (ST-1) vs. 10276 (ST-9) | <0.0001 |  | 6 (ST-1) vs. 9427 (ST-144) | <0.0001 |
| 7339 (ST-12) vs. 231 (ST-17) | 0.0232 |  | 104 (ST-1) vs. 9427 (ST-144) | <0.0001 |
| 229 (ST-23) vs. 231 (ST-17) | 0.0001 |  | 6 (ST-1) vs. 8422 (ST-291) | <0.0001 |
| 9427 (ST-144) vs. 231 (ST-17) | <0.0001 |  | 104 (ST-1) vs. 8422 (ST-291) | <0.0001 |
| 8422 (ST-291) vs. 231 (ST-17) | <0.0001 |  | 104 (ST-1) vs. 6 (ST-1) | 0.0067 |
| 229 (ST-23) vs. 9731 (ST-17) | 0.0006 |  | 123 (ST-498) vs. 211 (ST-28) | 0.0099 |
| 9427 (ST-144) vs. 9731 (ST-17) | <0.0001 |  | 123 (ST-498) vs. 104 (ST-1) | 0.0059 |
| 8422 (ST-291) vs. 9731 (ST-17) | <0.0001 |  |  |  |

**Table S3B: LDH for MOI of 20**

| **GBS isolates** | **Šidák's multiple comparisons test (adjusted p-value)** |  | **GBS isolates** | **Šidák's multiple comparisons test (adjusted p-value)** |
| --- | --- | --- | --- | --- |
| 231 (ST-17) vs. 203 (ST-8) | <0.0001 |  | 6 (ST-1) vs. 9731 (ST-17) | 0.04 |
| 9731 (ST-17) vs. 203 (ST-8) | <0.0001 |  | 123 (ST-498) vs. 9731 (ST-17) | <0.0001 |
| 211 (ST-28) vs. 203 (ST-8) | <0.0001 |  | 7339 (ST-12) vs. 211 (ST-28) | 0.0045 |
| 7339 (ST-12) vs. 203 (ST-8) | <0.0001 |  | 229 (ST-23) vs. 211 (ST-28) | <0.0001 |
| 9427 (ST-144) vs. 203 (ST-8) | 0.0264 |  | 9427 (ST-144) vs. 211 (ST-28) | <0.0001 |
| 6 (ST-1) vs. 203 (ST-8) | <0.0001 |  | 8422 (ST-291) vs. 211 (ST-28) | <0.0001 |
| 104 (ST-1) vs. 203 (ST-8) | <0.0001 |  | 6 (ST-1) vs. 211 (ST-28) | 0.0002 |
| 123 (ST-498) vs. 203 (ST-8) | 0.0008 |  | 123 (ST-498) vs. 211 (ST-28) | <0.0001 |
| 231 (ST-17) vs. 10276 (ST-9) | <0.0001 |  | 229 (ST-23) vs. 7339 (ST-12) | 0.0005 |
| 9731 (ST-17) vs. 10276 (ST-9) | <0.0001 |  | 9427 (ST-144) vs. 7339 (ST-12) | <0.0001 |
| 211 (ST-28) vs. 10276 (ST-9) | <0.0001 |  | 8422 (ST-291) vs. 7339 (ST-12) | <0.0001 |
| 7339 (ST-12) vs. 10276 (ST-9) | 0.001 |  | 104 (ST-1) vs. 7339 (ST-12) | 0.0001 |
| 9427 (ST-144) vs. 10276 (ST-9) | <0.0001 |  | 9427 (ST-144) vs. 229 (ST-23) | <0.0001 |
| 6 (ST-1) vs. 10276 (ST-9) | 0.0205 |  | 6 (ST-1) vs. 229 (ST-23) | 0.0084 |
| 104 (ST-1) vs. 10276 (ST-9) | <0.0001 |  | 104 (ST-1) vs. 229 (ST-23) | <0.0001 |
| 229 (ST-23) vs. 231 (ST-17) | <0.0001 |  | 8422 (ST-291) vs. 9427 (ST-144) | <0.0001 |
| 9427 (ST-144) vs. 231 (ST-17) | <0.0001 |  | 6 (ST-1) vs. 9427 (ST-144) | <0.0001 |
| 8422 (ST-291) vs. 231 (ST-17) | <0.0001 |  | 104 (ST-1) vs. 9427 (ST-144) | <0.0001 |
| 6 (ST-1) vs. 231 (ST-17) | 0.0047 |  | 123 (ST-498) vs. 9427 (ST-144) | <0.0001 |
| 123 (ST-498) vs. 231 (ST-17) | <0.0001 |  | 6 (ST-1) vs. 8422 (ST-291) | 0.0003 |
| 229 (ST-23) vs. 9731 (ST-17) | <0.0001 |  | 104 (ST-1) vs. 8422 (ST-291) | <0.0001 |
| 9427 (ST-144) vs. 9731 (ST-17) | <0.0001 |  | 104 (ST-1) vs. 6 (ST-1) | <0.0001 |
| 8422 (ST-291) vs. 9731 (ST-17) | <0.0001 |  | 123 (ST-498) vs. 104 (ST-1) | <0.0001 |

**Table S4: Statistical differences between individual isolates for OCR, as determined by one-way ANOVA with Šidák’s *post-hoc* test.**

| **GBS isolates** | **Šidák's multiple comparisons test (adjusted p-value)** |
| --- | --- |
| 8422 (ST-291)vs. 231 (ST-17) | 0.0269 |
| 123 (ST-498) vs. 231 (ST-17) | 0.0101 |
| 123 (ST-498) vs. 9731 (ST-17) | 0.034 |
| 8422 (ST-291) vs. 9427 (ST-144) | 0.0232 |
| 123 (ST-498) vs. 9427 (ST-144) | 0.0088 |
| 104 (ST-1) vs. 8422 (ST-291) | 0.0149 |
| 123 (ST-498) vs. 104 (ST-1) | 0.0019 |

**Table S5: Statistical differences between individual isolates for glyco ATP, as determined by one-way ANOVA with Šidák’s *post-hoc* test.**

| **GBS isolates** | **Šidák's multiple comparisons test (adjusted p-value)** |
| --- | --- |
| 229 (ST-23) vs. 9731 (ST-17) | 0.031 |
| 123 (ST-498) vs. 229 (ST-23) | 0.0127 |
| 123 (ST-498) vs. 9427 (ST-144) | 0.0403 |
| 123 (ST-498) vs. 6 (ST-1) | 0.0397 |
| 123 (ST-498) vs. 104 (ST-1) | 0.0145 |

**Table S6: Statistical differences between individual isolates for glyco ATP / OxPHOS ATP, as determined by one-way ANOVA with Šidák’s *post-hoc* test.**

| **GBS isolates** | **Šidák's multiple comparisons test (adjusted p-value)** |
| --- | --- |
| 231 (ST-17) vs. 203 (ST-8) | 0.0135 |
| 6 (ST-1) vs. 203 (ST-8) | 0.0483 |
| 7339 (ST-12) vs. 231 (ST-17) | 0.043 |

**Table S7: Statistical differences between individual isolates for ECAR / OCR, as determined by one-way ANOVA with Šidák’s *post-hoc* test.**

| **GBS isolates** | **Šidák’s multiple comparisons test (adjusted p-value)** |  | **GBS isolates** | **Šidák’s multiple comparisons test (adjusted p-value)** |
| --- | --- | --- | --- | --- |
| 9731 (ST-17) vs. 203 (ST-8) | 0.0208 |  | 7339 (ST-12) vs. 9731 (ST-17) | 0.0022 |
| 229 (ST-23) vs. 10276 (ST-9) | 0.0145 |  | 229 (ST-23) vs. 9731 (ST-17) | <0.0001 |
| 8422 (ST-291) vs. 10276 (ST-9) | 0.0352 |  | 9427 (ST-144) vs. 9731 (ST-17) | 0.0264 |
| 6 (ST-1) vs. 10276 (ST-9) | 0.0311 |  | 8422 (ST-291) vs. 9731 (ST-17) | <0.0001 |
| 211 (ST-28) vs. 231 (ST-17) | 0.033 |  | 6 (ST-1) vs. 9731 (ST-17) | <0.0001 |
| 229 (ST-23) vs. 231 (ST-17) | 0.0018 |  | 123 (ST-498) vs. 9731 (ST-17) | 0.0002 |
| 8422 (ST-291) vs. 231 (ST-17) | 0.0049 |  | 104 (ST-1) vs. 229 (ST-23) | 0.0197 |
| 6 (ST-1) vs. 231 (ST-17) | 0.0042 |  | 104 (ST-1) vs. 8422 (ST-291) | 0.0261 |
| 123 (ST-498) vs. 231 (ST-17) | 0.0107 |  | 104 (ST-1) vs. 6 (ST-1) | 0.0157 |
| 211 (ST-28) vs. 9731 (ST-17) | 0.0005 |  | 123 (ST-498) vs. 104 (ST-1) | 0.0145 |

**Table S8: Statistical differences between individual isolates for non-mitochondrial OCR, as determined by one-way ANOVA with Šidák’s *post-hoc* test..**

| **GBS isolates** | **Šidák's multiple comparisons test (adjusted p-value)** |  | **GBS isolates** | **Šidák's multiple comparisons test (adjusted p-value)** |
| --- | --- | --- | --- | --- |
| 8422 (ST-291) vs. 203 (ST-8) | 0.0036 |  | 8422 (ST-291) vs. 229 (ST-23) | 0.0485 |
| 123 (ST-498) vs. 203 (ST-8) | 0.0006 |  | 104 (ST-1) vs. 229 (ST-23) | 0.038 |
| 104 (ST-1) vs. 10276 (ST-9) | 0.0167 |  | 123 (ST-498) vs. 229 (ST-23) | 0.0093 |
| 8422 (ST-291) vs. 231 (ST-17) | 0.0015 |  | 8422 (ST-291) vs. 9427 (ST-144) | <0.0001 |
| 123 (ST-498) vs. 231 (ST-17) | 0.0003 |  | 123 (ST-498) vs. 9427 (ST-144) | <0.0001 |
| 8422 (ST-291) vs. 9731 (ST-17) | 0.0079 |  | 8422 (ST-291) vs. 229 (ST-23) | 0.039 |
| 123 (ST-498) vs. 9731 (ST-17) | 0.0013 |  | 104 (ST-1) vs. 8422 (ST-291) | <0.0001 |
| 9427 (ST-144) vs. 211 (ST-28) | 0.0069 |  | 104 (ST-1) vs. 6 (ST-1) | 0.0052 |
| 104 (ST-1) vs. 211 (ST-28) | 0.0011 |  | 123 (ST-498) vs. 6 (ST-1) | 0.0235 |
| 8422 (ST-291) vs. 7339 (ST-12) | 0.0096 |  | 123 (ST-498) vs. 104 (ST-1) | <0.0001 |
| 123 (ST-498) vs. 7339 (ST-12) | 0.0016 |  |  |  |
